# Association of *MTOR* and *AKT* Gene Polymorphisms with Susceptibility and Survival of Gastric Cancer

**DOI:** 10.1371/journal.pone.0136447

**Published:** 2015-08-28

**Authors:** Ying Piao, Ying Li, Qian Xu, Jing-wei Liu, Cheng-zhong Xing, Xiao-dong Xie, Yuan Yuan

**Affiliations:** 1 Tumor Etiology and Screening Department of Cancer Institute and General Surgery, the First Affiliated Hospital of China Medical University, and Key Laboratory of Cancer Etiology and Prevention (China Medical University), Liaoning Provincial Education Department, Shenyang, People’s Republic of China; 2 Oncology Department, General Hospital of Shenyang Military Region, Shenyang, Liaoning, People’s Republic of China; National Cancer Center, JAPAN

## Abstract

**Background:**

The phosphoinositide 3-kinase (PI3K)/protein kinase B (PKB, AKT)/mammalian target of rapamycin (mTOR) signaling pathway plays a critical role in angiogenesis and cell growth, proliferation, metabolism, migration, differentiation, and apoptosis. Genetic diversity in key factors of this pathway may influence protein function and signal transduction, contributing to disease initiation and progression. Studies suggest that *MTOR* rs1064261 and *AKT* rs1130233 polymorphisms are associated with risk and/or prognosis of multiple cancer types. However, this relationship with gastric cancer (GC) remains unclear. The aim of this study was to investigate the role of *MTOR* and *AKT* polymorphisms in the risk and prognosis of GC.

**Methods:**

The Sequenom MassARRAY platform was used to genotype 1842 individuals for *MTOR* rs1064261 T→C and *AKT* rs1130233 G→A polymorphisms. ELISA was used to detect *Helicobacter pylori* antibodies in serum. Immunohistochemical analysis was used to detect total and phosphorylated MTOR and AKT proteins.

**Results:**

The *MTOR* rs1064261 (TC+CC) genotype and the *AKT* rs1130233 (GA+AA) genotype were associated with increased risk of GC in men (*P* = 0.049, *P* = 0.030). In *H*. *pylori*-negative individuals, the *AKT* rs1130233 GA and (GA+AA) genotypes were related to increased risk of atrophic gastritis (AG; *P* = 0.012, *P* = 0.024). Notably, the *AKT* rs1130233 (GA+AA) genotype demonstrated significant interactions with *H*. *pylori* in disease progression from healthy controls (CON) to AG (*P* = 0.013) and from AG to GC (*P* = 0.049). Additionally, for individuals with the *AKT* rs1130233 variant, those in the H. pylori-positive group had higher levels of phosphorylated AKT (p-AKT) expression. The *AKT* rs1130233 genotype was found to be associated with clinicopathological parameters including lymph node metastasis and alcohol drinking (*P*<0.05).

**Conclusion:**

*MTOR* rs1064261and *AKT* rs1130233 polymorphisms were associated with increased GC risk in males and increased AG risk in *H*. *pylori*-negative individuals. A significant interaction existed between the *AKT* rs1130233 genotype and *H*. *pylori* infection in CON→AG→GC disease progression. The *AKT* rs1130233 genotype influenced p-AKT protein expression in *H*. *pylori*-infected individuals.

## Introduction

Gastric cancer (GC) is one of the most common cancers worldwide and a leading cause of cancer-related death [1]. The initiation and development of GC is a multistep process influenced by genetic and environmental factors. Studies have indicated that exogenous environmental factors including *Helicobacter pylori* (HP) infection, diet habits, smoking, alcohol drinking, and economic factors contribute to gastric carcinogenesis [2]. Under similar environmental conditions, individuals with different genetic background suffer different risks of cancer with diverse clinical outcomes. Until now, the interaction of genetic and environmental factors with gastric carcinogenesis has been largely unknown.

Polymorphisms are a class of genetic factors that participate in gastric carcinogenesis and determine inter-individual variations in GC risk. Genetic polymorphisms can weaken intrinsic protective mechanisms and increase the damage caused by environmental carcinogens. Carriers of susceptible genotypes are at a greater risk of developing cancer than those with resistant genotypes under similar conditions. Therefore, genetic factors play a crucial role in GC risk and clinical outcome.

The phosphoinositide 3-kinase (PI3K)/protein kinase B (PKB, AKT)/mammalian target of rapamycin (MTOR) pathway regulates various cellular functions including growth, proliferation, migration, and apoptosis. Tapia et al. reported that the PI3K/AKT/MTOR pathway is activated in GC and that most proteins (phosphorylated and unphosphorylated forms) studied so far in this pathway are overexpressed in tumor tissues [3]. MTOR is a key member of the PI3K/AKT/MTOR pathway and a core metabolic signaling molecule [4, 5]. Single nucleotide polymorphisms (SNPs) may affect MTOR expression and transcriptional activity, thereby altering protein function. So far, a total of 14 polymorphisms in this gene have been studied, of which three SNPs (rs2295080, rs1883965, and rs2536) have been most widely reported. The rs2295080 polymorphism in the promoter region is reported to reduce the risk of renal cell carcinoma [6], GC [7], and prostate cancer [8] by downregulating endogenous protein expression. The rs1883965 polymorphism has been associated with an increased risk of GC [9] and esophageal squamous cancer [10]. Moreover, rs2536 polymorphism is related to a significantly increased risk of prostate cancer [8]. In addition, the *MTOR* rs11121704 polymorphism is associated with worse clinical parameters (death, metastasis, and chemotherapy resistance) [11]. There are 45 exons in the *MTOR* gene, and the rs1064261 polymorphism is located in exon 18. The T→C variation is located at the boundary of exon 18 and intron 19, but its relation with disease is still unclear.

The *AKT* gene is critical for cell survival and encodes an important downstream effector of the PI3K/*AKT*/*MTOR* pathway that regulates key cellular functions including glucose metabolism and protein synthesis. *AKT*, alternatively known as *AKT1*, has five widely studied polymorphisms (rs3803300, rs1130214, rs2494732, rs2498804, and rs1130233); the first four have been linked to the risk or prognosis of nasopharyngeal carcinoma, oral squamous cell carcinoma, and non-small cell lung cancer. The rs1130233 polymorphism is located in exon 8 and the G→A variation is located at the boundary of exon 8 and intron 7. The *AKT*1 AA haplotype for both rs1130233 and rs2494732 is reported to confer an elevated risk of nasopharyngeal carcinoma [12]; the haplotype containing variant alleles of rs1130214 and rs3803300 polymorphisms significantly increases susceptibility to oral squamous cell carcinoma [13]. Carriers of the (GT+GG) genotype of *AKT*1 rs2498804 or the CT/TT genotype of *AKT*1 rs2494732 were found to have an increased risk of brain metastasis of non-small cell lung cancer [14]. *AKT*1 rs3803300, rs1130214, and rs2494732 have significant effects on survival in non-small cell lung cancer patients: patients with the rs3803300 G allele and rs1130214 G allele had shorter overall survival (OS) and disease-free survival (DFS) times [15].

Although variations in *MTOR* and *AKT* play important roles in gastric carcinogenesis, no study has investigated the relation of *AKT* polymorphism with GC risk and prognosis. In addition, the mechanistic link between *MTOR* rs1064261 and both cancer susceptibility and survival are unknown. To elucidate whether *MTOR* and *AKT* polymorphisms can serve as risk and/or prognostic markers for GC, we investigated *MTOR* rs1064261 and *AKT* rs1130233 polymorphisms in relation to GC risk and their interactions with *H*. *pylori* in a case–control study of 1842 subjects. In 205 individuals with sufficient data, clinicopathological parameters were analyzed to explore the association of these two polymorphisms with GC prognosis and thus identify new biomarkers for GC risk and prognosis.

## Materials and Methods

### Study design and study population

The design of this study was approved by the Human Ethics Committee of the First Affiliated Hospital of China Medical University (Shenyang, China). Each participant provided written informed consent during an epidemiological investigation. The study design included two parts (polymorphism and protein level analyses) and associations between polymorphisms and disease risk and prognosis were investigated. In the GC risk analysis, the GC patients were from the First Affiliated Hospital of China Medical University which obtained surgery operation resection or gastroscopic diagnosis/treatment between 2004 and 2013. The AG patients and controls were recruited from a health check program in Zhuanghe, Liaoning Province, between 2002 and 2013. All diagnoses were based on gastroscopic and histopathological examinations.

In addition, GC cases were classified into intestinal type and diffuse type based on Lauren’s classification [16, 17]; AG classification and staging were based on the new Sydney system [18, 19]. Control participants had normal stomach findings or gastritis only. The information about the smoking habit, alcohol consumption and family history were acquired by “face to face” questionnaire survey. The smokers and alcoholic drinks were defined as follow: if one smoked more than once per day and if this lasted more than 1 year, then this individual was defined as smokers, and this situation included current smokers and former smokers who had quit smoking for more than 1 year. Persons who did not satisfy this situation were defined as never smokers. If one consumed one bottle of beer or a fifth pound of liquor a day and if this situation lasted more than 1 year, then this person was considered drinkers. Individuals who did not fit this standard were defined as nondrinkers. Fasting venous blood was obtained from each participant and stored at −20°C as serum and clotted cells.

To further evaluate the relation of polymorphisms with clinicopathological parameters and survival of GC, we performed a prognostic analysis of GC patients for whom sufficient clinical data was available. Histology data were assessed according to Health Organization criteria and tumor–node–metastasis (TNM) staging of postoperative pathologic specimens was done according to 7th edition of the of the International Union Against Cancer (UICC)/American Joint Committee on Cancer (AJCC) (2010) criteria. Patients with distant metastasis before surgery, those who received radiotherapy or chemotherapy before surgery, and those with insufficient information for prognostic analysis were excluded. A sensitivity analysis was performed to test whether the excluded patients had effect to the survival analysis. Follow-up for all patients was completed in May 2014.

### SNP genotyping

Genomic DNA was extracted from blood samples using the phenol–chloroform method [20] and diluted to working concentration of 50 ng/μl for *MTOR* rs1064261 T→C and *AKT* rs1130233 G→A genotyping. Samples were placed randomly in 384-well plates and analyzed in a blinded manner to disease status. Genotyping was performed using the Sequenom MassARRAY platform (Sequenom, San Diego, California, USA) according to the manufacturer’s instructions. For quality control, 5% of samples underwent repeated genotyping; the results were 100% consistent.

### H. pylori serology examination

Serology analysis to detect *H*. *pylori* infection was performed using ELISA (*H*. *pylori*-IgG ELISA kit, BIOHIT, Helsinki, Finland), as described previously [21]. A reading of >34 enzyme immune units was regarded to indicate *H*. *pylori* positivity.

### Detection of MTOR and AKT proteins in tissue

Immunohistochemical analysis was used to determine the expression of total and phosphorylated MTOR and AKT (p-MTOR and p-AKT) proteins in 65 formalin-fixed, paraffin-embedded GC tissue samples. Tissue samples were cut into 4-μm-thick sections and mounted onto poly-l-lysine-coated glass slides. After citric acid antigen retrieval, primary antibody was incubated with tissue sections at 4°C overnight (dilution concentration: MTOR and p-MTOR 1:100, AKT 1:300, and p-AKT 1:50, all were purchased from Cell Signaling Technology). After three 5-min washes in phosphate buffer saline, tissue sections were incubated with biotinylated secondary antibody (Maixin, Fujian, China) and streptavidin–biotin peroxidase for 10 min each at 37°C. For negative controls, primary antibody was replaced with PBS buffer.

### Statistical analysis

Statistical analysis was performed using SPSS (version 18.0) statistical software (SPSS, Chicago, IL, USA). Hardy–Weinberg equilibrium (HWE) was first evaluated in healthy controls. Adjusted odds ratios and 95% confidence intervals (CIs) for the relation between both polymorphisms and disease risk were calculated by multivariable logistic regression, with adjustment for gender, age, and *H*. *pylori* infection status. In a stratified analysis, when stratified by age, the sex and *H*.*pylori* infection status were adjusted; when stratified by sex, the age and *H*.*pylori* infection status were adjusted; and when stratified by *H*.*pylori* infection status, the sex and age were adjusted. The likelihood ratio test was performed to assess the interaction effects of genotype and *H*. *pylori* on disease risk by comparing the model involving only the main effects of gender, age, *H*. *pylori* status, and genotype with the full model (also containing the interaction term of genotype with *H*. *pylori* status). Pearson’s χ^2^ test was used to evaluate the relation of different genotypes with the clinicopathological parameters of GC. Fisher’s exact method was used when the expected frequency was less than five. The Kaplan–Meier method was used to visualize OS by genotype group. The log-rank test was used to investigate differences in survival distributions. Univariable and multivariable Cox proportional hazards models were performed to calculate the crude or adjusted hazards ratios and 95% CIs for each genotype to estimate the effect on survival with or without adjustment for confounding factors. Logistic regression analysis was used to explore the effect of polymorphisms on protein expression adjusted by gender, age, and *H*. *pylori* status. Two-tailed *P* values of <0.05 were considered statistically significant.

## Results

### Baseline patient characteristics

The demographic and clinical characteristics of the 1842 individuals involved in the risk analysis comprising 483 GC patients, 686 AG patients and 673 healthy controls are shown in Table 1. Both SNPs were present in HWE; the genotype distribution is listed in Table 2. The result of the sensitivity analysis was shown in S1 Table, which verified the excluded patients had no effect to the survival analysis. The demographic and clinical characteristics of the 205 GC patients involved in the survival analysis including age, sex, Borrmann classification, Lauren’s classification, TNM stage, growth pattern, invasion depth, lymph node metastasis, smoking, drinking, family history, and *H*. *pylori* infection are shown in Table 3.

**Table 1:** The basic messages of the objects.

Variability	Gastric mucosa status				
	CON	AG	GC		
				Intestinal-type GC	Diffuse-type GC
**For SNP study**					
N	673	683	461	138	200
Age	***P*<0.001**				
Mean±SD	52.93±9.90	55.03±9.20	58.95±11.24	60.54±10.56	58.21±12.50
Median	53	56	58	59	58
Range	17–85	16–82	26–87	31–84	26–87
Gender	***P*<0.001**				
Male	342(50.8)	386(56.3)	316(68.0)	107(76.4)	125(61.9)
Female	331(49.2)	300(43.7)	149(32.0)	33(23.6)	77(38.1)
*H*.*pylori*	***P<*0.001**				
Positive	146(21.8)	403(59.6)	249(52.1)	78(56.1)	105(50.2)
Negative	523(78.2)	273(40.4)	229(47.9)	61(43.9)	104(49.8)
**For protein expression study**					
N			65		
Age					
Mean±SD			55.9±11.4		
Median			56		
Range			31–82		
Gender					
Male			42(64.6)		
Female			23(35.4)		
*H*.*pylori*					
Positive			25(38.5)		
Negative			40(61.5)		

**Abbreviations:** CON, control; AG, atrophic gastritis; GC, gastric cancer; SD, standard deviation; N, number of the objects.

**Table 2:** Association of mTOR rs1064261 and AKT rs1130233 polymorphisms with the risk of atrophic gastritis and gastric cancer.

Stratified	SNP	Gastric mucosa status			AG vs. CON		GC vs. AG		GC vs. CON		GC vs. CON+AG	
		CON(%)	AG(%)	GC(%)	OR(95%CI)	*P-*value	OR(95%CI)	*P-*value	OR(95%CI)	*P*-value	OR(95%CI)	*P*-value
	mTOR rs1064261*											
	TT	560(83.2)	584(85.1)	402(83.2)	1(Ref)		1(Ref)		1(Ref)		1(Ref)	
	TC	107(15.9)	97(14.1)	76(15.7)	0.92(0.66–1.28)	0.626	1.22(0.87–1.71)	0.259	0.96(0.67–1.37)	0.804	1.12(0.83–1.51)	0.478
	CC	6(0.9)	5(0.7)	5(1.0)	0.56(0.15–2.09)	0.391	1.78(0.50–6.36)	0.374	1.26(0.35–4.51)	0.722	1.38(0.46–4.15)	0.564
	HWE	0.724										
	AKT rs1130233*											
	GG	144(21.5)	129(19.1)	89(18.5)	1(Ref)		1(Ref)		1(Ref)		1(Ref)	
	GA	329(49.0)	362(53.5)	236(49.0)	1.21(0.89–1.64)	0.234	1.06(0.76–1.49)	0.719	1.14(0.80–1.62)	0.481	1.16(0.86–1.56)	0.347
	AA	198(29.5)	186(27.5)	157(32.6)	1.09(0.77–1.54)	0.637	1.26(0.88–1.82)	0.214	1.25(0.85–1.85)	0.252	1.31(0.95–1.82)	0.100
	HWE	0.737										
Sex	mTOR rs1064261^§^											
Male	TT	283(82.7)	336(87.0)	260(82.3)	1(Ref)		1(Ref)		1(Ref)		1(Ref)	
	TC	55(16.1)	48(12.4)	54(17.1)	0.70(0.44–1.12)	0.138	**1.55(1.00–2.38)**	**0.049**	0.97(0.62–1.51)	0.880	1.29(0.89-.186)	0.184
	CC	4(1.2)	2(0.5)	2(0.6)	0.28(0.04–1.94)	0.199	1.34(0.18–9.79)	0.775	0.73(0.12–4.30)	0.724	0.87(0.17–0.448)	0.863
Sex	AKT rs1130233^§^											
Male	GG	74(21.7)	84(22.0)	53(16.8)	1(Ref)		1(Ref)		1(Ref)		1(Ref)	
	GA	170(49.9)	200(52.5)	154(48.7)	0.97(0.64–1.47)	0.891	1.32(0.87–2.00)	0.192	1.30(0.83–2.05)	0.255	1.33(0.91–1.94)	0.139
	AA	97(28.4)	97(25.5)	109(34.5)	0.88(0.55–1.42)	0.609	**1.76(1.12–2.77)**	**0.014**	**1.70(1.03–2.81)**	**0.039**	**1.71(1.14–2.57)**	**0.009**
*H*.*pylori*	AKT rs1130233^#^											
Negative	GG	118(22.6)	43(15.9)	47(20.6)	1(Ref)		1(Ref)		1(Ref)		1(Ref)	
	GA	246(47.2)	149(55.2)	105(46.1)	**1.69(1.12–2.54)**	**0.012**	0.72(0.43–1.19)	0.194	1.21(0.78–1.87)	0.400	1.02(0.67–1.53)	0.945
	AA	157(30.1)	78(28.9)	76(33.3)	1.39(0.89–2.18)	0.144	0.94(0.55–1.62)	0.830	1.29(0.81–2.05)	0.288	1.18(0.76–1.83)	0.470

**Note:**

*using Logistic Regession adjusted by sex, age and *H*.*pylori* infection status.

^§^using Logistic Regession adjusted by age and *H*.*pylori* infection status.

^#^using Logistic Regession adjusted by sex and age.

**Abbreviations:** SNP, single nucleotide polymorphism; CON, control; AG, atrophic gastritis; GC, gastric cancer; OR, odds ratio; CI, confidence interval; Ref, reference; HWE, Hardy-Weinberg Equilibrium in population.

**Table 3:** Clinical features of gastric cancer patients.

Variables	GC patients	Death	MST	Log-rank	HR(95%CI)
	N = 205(%)	N = 67	M	*P-value*	
Age					
≤50	54(26.34)	20	55.31^a^		1(Ref)
>50	151(73.66)	47	62.23^a^	0.495	0.83(0.49–1.41)
Sex					
Female	63(30.73)	25	43.43^a^		1(Ref)
Male	142(69.27)	42	64.93^a^	0.130	0.66(0.42–1.20)
Borrmann type					
Borrmann I–II	76(37.07)	29	60.81^a^		1(Ref)
Borrmann III–IV	101(49.27)	37	44.0	0.632	1.13(0.69–1.84)
Lauren classification					
Intestinal	75(36.59)	18	64.55^a^		1(Ref)
Diffuse	129(62.93)	49	57.88^a^	**0.036**	**1.78(1.04–3.06)**
TNM stage					
I–II	120(58.54)	22	73.69^a^		1(Ref)
III–IV	85(41.46)	45	31.0	**4.01×10** ^**−7**^	**3.76(2.25–6.27)**
Growth pattern					
Massive and Nested	70(34.15)	12	35.07^a^		1(Ref)
Diffused	64(31.22)	24	27.15^a^	**0.005**	**2.73(1.36–5.46)**
Depth of invasion					
T1+T2	43(20.98)	1	37.63^a^		1(Ref)
T3+T4	91(44.39)	35	28.74^a^	**0.003**	**20.15(2.76–147.11)**
Lymphatic metastasis					
Negative	77(37.56)	11	71.77^a^		1(Ref)
Positive	128(62.44)	56	53.01^a^	**4.39×10** ^**−5**^	**3.86(2.02–7.36)**
Smoking					
Never Smoker	80(39.02)	21	31.79^a^		1(Ref)
Ever Smoker	54(26.34)	15	31.23^a^	0.795	1.09(0.56–2.12)
Alcohol drinking					
Nondrinker	90(43.90)	24	31.59^a^		1(Ref)
Drinker	44(21.46)	12	30.76^a^	0.909	1.04(0.52–2.08)
Family history					
No	107(52.20)	33	30.48^a^		1(Ref)
Yes	27(13.17)	3	35.85^a^	0.052	0.31(0.10–1.01)
*H*. *pylori*-IgG					
Negative	86(41.95)	29	60.74^a^		1(Ref)
Positive	117(57.07)	37	59.02^a^	0.743	0.92(0.57–1.50)

**Note:**

^a^, mean survival time was provided when MST could not be calculated.

**Abbreviations:** MST, median survival time (months); HR, hazard rate; CI, confidence interval; GC, gastric cancer; N, number of patients; M, months.

### Association of SNPs with GC and AG risk

Multivariable logistic regression was used to investigate the association of *MTOR* rs1064261and *AKT* rs1130233 with GC and AG risk. There was no significant difference of *MTOR* rs1064261 and *AKT* rs1130233 on the risk of CON→AG, AG→GC, CON→GC, or CON+AG→GC progression (Table 2).

In the stratification analysis of males (Table 2), carriers of the TC genotype of *MTOR* rs1064261 had a 1.55-fold increased risk of GC compared with AG (*P* = 0.049, 95% CI 1.00–2.38); the A allele of *AKT* rs1130233 conferred 1.32-fold, 1.29-fold, and 1.30-fold increases in AG→GC, CON→GC, and (CON+AG)→GC progression, respectively, over the G allele (*P* = 0.013, 95% CI 1.06–1.64; *P* = 0.038, 95% CI 1.01–1.64; and *P* = 0.008, 95% CI 1.07–1.58, S2 Table). In the *H*. *pylori*-negative group, the *AKT* rs1130233 GA genotype was associated with a 1.69-fold increased risk of AG compared with CON (*P* = 0.012, 95% CI 1.12–2.54, Table 2). In addition, no significant association was found between these two polymorphisms and intestinal-type or diffuse-type GC in the overall and stratified analyses (S3 and S4 Tables).

We then performed an interaction analysis for *MTOR* rs1064261 and *AKT* rs1130233 with *H*. *pylori* infection. The results indicated that the *AKT* rs1130233 (GA+AA) genotype had a significant interaction with *H*. *pylori* infection in CON→AG and AG→GC progression (*P* = 0.013 and *P* = 0.049; Table 4). However, no significant interaction of *MTOR* rs1064261 with *AKT* rs1130233 was observed for CON→AG→GC progression in the overall and stratified analyses (S5 Table). Additionally, there was no significant interaction between these two polymorphisms and *H*. *pylori* infection in CON→AG→GC progression (Table 4).

**Table 4:** The interaction of MTOR rs1064261 and AKT rs1130233 polymorphisms in the risk of atrophic gastritis and gastric cancer*.

Genotypes			AG vs. CON		GC vs. AG		GC vs. CON		GC vs. CON+AG	
			*H*.*pylori*(-)	*H*.*pylori*(+)	*H*.*pylori*(-)	*H*.*pylori*(+)	*H*.*pylori*(-)	*H*.*pylori*(+)	*H*.*pylori*(-)	*H*.*pylori*(+)
	MTOR rs1064261									
	TT	Controls/Cases	432/229	124/348	229/196	348/202	432/196	124/202	661/196	472/202
		OR(95%CI)	1(Ref)	5.33(4.10–6.91)	1(Ref)	0.68(0.53–0.89)	1(Ref)	3.64(2.74–4.83)	1(Ref)	1.46(1.16–1.84)
	TC+CC	Controls/Cases	91/44	22/55	44/33	55/47	91/33	22/47	135/33	77/47
		OR(95%CI)	0.92(0.62–1.37)	4.76(2.83–8.00)	0.92(0.56–1.50)	1.00(0.65–1.56)	0.85(0.55–1.31)	4.78(2.79–8.18)	0.87(0.58–1.32)	2.08(1.39–3.11)
			*P*_interaction_ = 0.926		*P*_interaction_ = 0.195		*P*_interaction_ = 0.270		*P*_interaction_ = 0.093	
	AKT rs1130233									
	GG	Controls/Cases	118/43	24/86	43/47	86/41	118/47	24/41	161/47	110/41
		OR(95%CI)	1(Ref)	9.83(5.55–17.41)	1(Ref)	0.43(0.24–0.76)	1(Ref)	4.23(2.27–7.87)	1(Ref)	1.26(0.76–2.08)
	GA+AA	Controls/Cases	403/227	122/313	227/181	313/208	403/181	122/208	630/181	435/208
		OR(95%CI)	1.54(1.05–2.26)	7.02(4.67–10.54)	0.77(0.49–1.24)	0.64(0.41–1.02)	1.19(0.81–1.76)	4.52(2.99–6.85)	1.04(0.72–1.52)	1.73(1.19–2.53)
			***P***_**interaction**_ **= 0.013**		***P***_**interaction**_ **= 0.049**		*P*_interaction_ = 0.702		*P*_interaction_ = 0.366	
MTOR rs1064261	AKT rs1130233									
TT	GG	Controls/Cases	35/92	75/20	5/8	9/11	42/92	32/20	42/127	*32/95*
		OR(95%CI)	1(Ref)	9.86(5.26–18.48)	1(Ref)	0.36(0.19–0.67)	1(Ref)	3.51(1.77–6.96)	1(Ref)	1.02(0.59–1.77)
TT	GA+AA	Controls/Cases	192/339	269/104	28/153	38/170	153/339	170/104	153/531	170/373
		OR(95%CI)	1.48(0.97–2.27)	6.77(4.32–10.63)	0.71(0.43–1.18)	0.56(0.34–0.93)	1.05(0.69–1.61)	3.82(2.43–5.99)	0.93(0.62–1.39)	1.47(0.98–2.21)
TC+CC	GG	Controls/Cases	8/26	11/4	8/35	11/75	5/26	9/4	5/34	9/15
		OR(95%CI)	0.81(0.34–1.96)	7.23(2.16–24.21)	0.58(0.17–1.93)	0.67(0.24–1.86)	0.47(0.17–1.30)	4.84(1.38–17.04)	0.49(0.18–1.34)	1.78(0.70–4.52)
TC+CC	GA+AA	Controls/Cases	35/64	44/18	35/192	44/269	28/64	38/18	28/99	38/62
		OR(95%CI)	1.44(0.82–2.53)	6.43(3.28–12.59)	0.74(0.38–1.45)	0.78(0.41–1.46)	1.06(0.59–1.90)	4.98(2.53–9.81)	0.95(0.54–1.65)	1.99(1.16–3.44)
			*P*_interaction_ = 0.809		*P*_interaction_ = 0.258		*P*_interaction_ = 0.432		*P*_interaction_ = 0.260	

**Note:**

*using Logistic Regession adjusted by sex and age.

**Abbreviations:** CON, control; AG, atrophic gastritis; GC, gastric cancer; OR, odds ratio; CI, confidence interval; Ref, reference.

### Associations of SNPs with clinicopathological parameters of GC patients

Analysis of the relation of *MTOR* rs1064261 and *AKT* rs1130233 with clinicopathological parameters in GC patients suggested that the *AKT* rs1130233 GA and (GA+AA) variant genotypes occur more frequently in GC patients without lymph node metastasis than in those with lymph node metastasis (GA 89.1% vs. 71.1%, *P* = 0.012; GA+AA 92.2% vs. 81.1%, *P* = 0.030). The GA, AA, and (GA+AA) genotypes were more frequent in drinkers than in nondrinkers (GA 61.0% vs. 95.8%, *P* = 0.047; AA 63.6% vs. 88.9%, *P* = 0.047; GA+AA 76.8% vs. 96.9%, *P* = 0.038; Table 5). However, no significant association was observed between *MTOR* rs1064261 variants and clinicopathological parameters in GC patients.

**Table 5:** Correlation between MTOR rs1064261 or AKT rs1130233 polymorphisms and clinicopathological parameters in gastric cancer.

Variables	Wild	Heterozygous	*P*	Mutation	*P*	Dominance model	Recessive model
**MTOR rs1064261**							
Age			0.772		1.000^a^	0.845	1.000^a^
≤50	44	10		0			
>50	124	25		1			
Sex			0.278		0.325^a^	0.400	0.309^a^
Male	114	27		0			
Female	54	8		1			
Borrmann type			0.814		0.429^a^	0.672	0.432^a^
Borrmann I–II	62	13		1			
Borrmann III–IV	84	16		0			
Lauren classification			0.443		1.000^a^	0.381	1.000^a^
Intestinal	64	11		0			
Diffuse	105	24		1			
TNM stage			0.855		1.000^a^	0.759	1.000^a^
I–II	98	21		1			
III–IV	70	14		0			
Growth pattern			0.787		NA	0.787	NA
Massive and Nested	59	11		0			
Diffused	52	11		0			
Lymphatic metastasis			0.266		0.361^a^	0.196	0.377^a^
Negative	60	16		1			
Positive	108	19		0			
Depth of invasion			0.955		NA	0.955	NA
T1+T2	36	7		0			
T3+T4	75	15		0			
Smoking			0.557		NA	0.557	NA
Nonsmoker	68	12		0			
Smoker	43	10		0			
Acohol drinking			0.150		NA	0.150	NA
Nondrinker	78	12		0			
Drinker	33	10		0			
Family history			0.774^a^		NA	0.774^a^	NA
No	89	17		0			
Yes	22	5		0			
*H*. *pylori*-IgG			0.171		0.443^a^	0.241	0.421^a^
Negative	73	11		1			
Positive	93	24		0			
**AKT rs1130233**							
Age			0.627		0.799	0.673	0.873
≤50	7	30		17			
>50	23	78		49			
Sex			0.733		0.209	0.458	0.156
Male	19	72		50			
Female	11	36		16			
Borrmann type			0.960		0.880	0.922	0.866
Borrmann I–II	11	39		26			
Borrmann III–IV	15	52		33			
Lauren classification			0.758		0.594	0.973	0.262
Intestinal	11	36		28			
Diffuse	19	71		38			
TNM stage			0.912		0.618	0.887	0.391
I–I	18	66		36			
III–IV	12	42		30			
Growth pattern			0.957		0.624	0.810	0.497
Massive and Nested	9	36		25			
Diffused	9	35		19			
Lymphatic metastasis			**0.012**		0.183	**0.030**	0.369
Negative	6	49		22			
Positive	24	59		44			
Depth of invasion			0.970		0.769	0.922	0.629
T1+T2	6	24		13			
T3+T4	12	47		31			
Smoking			0.560		0.578	0.544	0.861
Nonsmoker	12	42		26			
Smoker	6	29		18			
Alcohol drinking			**0.047**		**0.047**	**0.038**	0.484
Nondrinker	16	25		28			
Drinker	2	46		16			
Family history			0.083^a^		0.541^a^	0.203^a^	0.343
No	12	61		33			
Yes	6	10		11			
*H*. *pylor*i-IgG			0.973		0.759	0.880	0.680
Negative	13	46		26			
Positive	17	61		39			

**Note:**

^a^, when the theoretical frequency of less than 5, using the Fisher's exact test; Wild, heterozygous, mutation, dominance model and recessive model of MTOR rs1064261 polymorphisms are TT, TC, CC, TC+CC vs. TT and CC vs. TC+TT, respectively. Wild, heterozygous, mutation, dominance model and recessive model of AKT rs1130233 polymorphisms are GG, GA, AA, GA+AA vs. GG, AA vs. GA+GG, respectively.

**Abbreviations:** NA, not available; T1+T2, mucosa, submucosa, muscularis propria; T3+T4, subserosa, serosa.

### No association between SNPs and GC patient survival

Univariable and multivariable Cox proportional hazards models were performed to assess the effect of *MTOR* rs1064261 and *AKT* rs1130233 polymorphisms on GC survival (Table 6). Because Lauren’s classification, TNM stage, growth pattern, invasion depth, and lymph node metastasis were significantly associated with survival (*P*<0.05, shown in Table 3), they were considered as adjusted covariables in the Cox proportional hazards regression model. The results showed no significant association between the two SNPs and GC prognosis (Table 6). Similarly, a stratified analysis did not demonstrate any significant association between SNP genotype and GC prognosis (S6 Table).

**Table 6:** Univariable and multivariable cox proportional hazard analysis for MTOR rs1064261 and AKT rs1130233 polymorphisms.

Variables	All GC	Death	MST	Univariable		Multivariable^b^	
	N	N	M	*P-value*	HR(95%CI)	*P-value*	HR(95%CI)
MTOR rs1064261							
TT	168	57	NA		1(Ref)		1(Ref)
TC	35	10	NA	0.593	0.83(0.43–1.63)	0.224	0.52(0.18–1.49)
CC	1	0	NA	0.655	0.05(0–27266.94)	NA	NA
TC+CC vs. TT				0.523	0.80(0.41–1.57)	0.224	0.52(0.18–1.49)
CC vs. TC+TT				0.663	0.05(0–36725.40)	NA	NA
AKT rs1130233							
GG	30	10	62.81^a^		1(Ref)		1(Ref)
GA	108	33	59.47^a^	0.893	0.95(0.47–1.93)	0.517	1.44(0.48–4.35)
AA	66	24	54.30^a^	0.790	1.11(0.53–2.31)	0.434	1.57(0.51–4.86)
GA+AA vs. GG				0.991	1.00(0.51–1.97)	0.483	1.45(0.51–4.15)
AA vs. GG+GA				0.546	1.17(0.71–1.92)	0.882	1.05(0.54–2.07)

**Note:**

^a^, mean survival time was provided when MST could not be calculated;

^b^, using multivariable COX proportional hazards model adjusted by Lauren classification, TNM stage, growth pattern, depth of invasion, lymphatic metastasis, and multivariate survival analysis was carried out by adding the SNP variable to the clinicopathological parameters with *P*<0.05.

**Abbreviations:** MST, median survival time (months); HR, hazard rate; CI, confidence interval; Ref, reference; GC, gastric cancer; NA, not available; N, number; M, months.

### SNP genotype correlates with total and phosphorylated proteins expression

**Fig 1:**
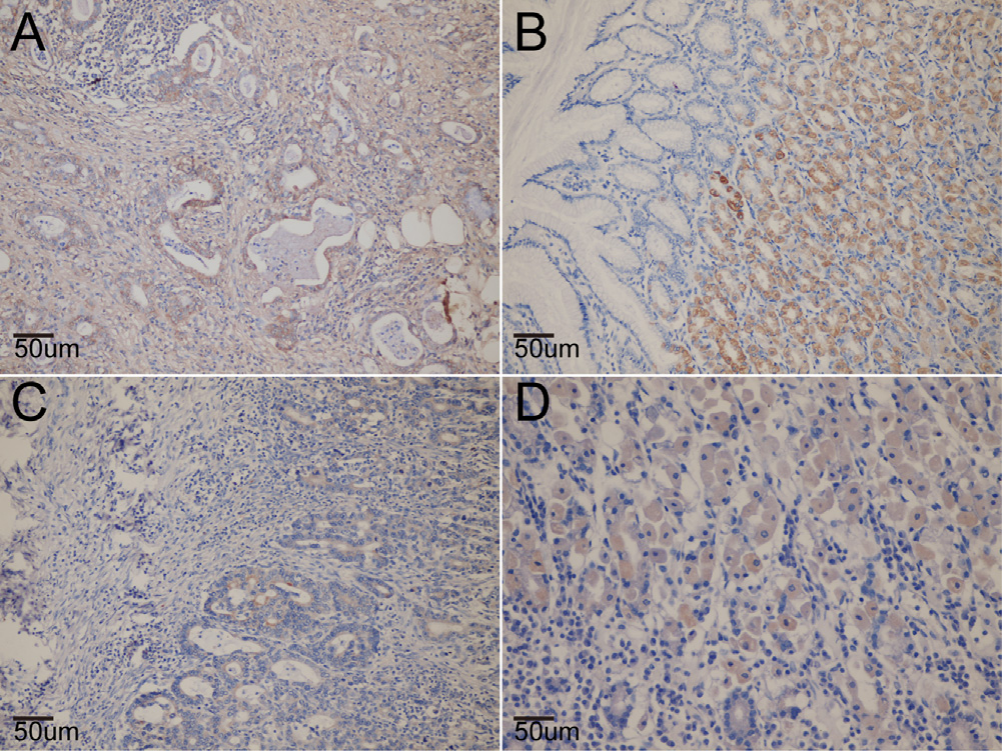
The expression of mTOR and AKT protein in tissue in situ. A. the expression of mTOR protein in gastric cancer mucosa (100×); B. the expression of p-mTOR protein in gastric mucosa (100×); C. the expression of AKT protein in gastric cancer mucosa (100×); D. the expression of p-AKT protein in gastric mucosa (100×).

The expression of total and phosphorylated MTOR and AKT proteins was analyzed in tissues from the different groups of participants (Fig 1). Overall, there was no significant association between the *MTOR* rs1064261 or *AKT* rs1130233 polymorphism and total or phosphorylated MTOR and AKT proteins. In the *H*. *pylori*-positive subgroup, the proportion of p-AKT-positive cells was significantly higher in the variant genotype group than in the wild-type group (*P* = 0.045; Table 7).

**Table 7:** The effect on the polymorphisms to its protein expression in the group of gastric cancer *

	Protein Expression		*P-*value
Variable	Negative	Positive	
**MTOR**			
MTOR rs1064261 TT	14(82.4)	9(75.0)	
MTOR rs1064261 TC	3(17.6)	3(25.0)	0.645
MTOR rs1064261 CC	0	0	NA
**Phosphoryltion MTOR**			
MTOR rs1064261 TT	30(83.3)	11(73.3)	
MTOR rs1064261 TC	6(16.7)	4(26.7)	0.126
MTOR rs1064261 CC	0	0	NA
**AKT(PAN)**			
AKT rs1130233 GG	1(5.9)	1(12.5)	
AKT rs1130233 GA	8(47.1)	5(62.5)	0.475
AKT rs1130233 AA	8(47.1)	2(25.0)	0.266
GA+AA vs. GG			0.638
AA vs. GA+GG			0.236
**Phosphoryltion AKT**			
AKT rs1130233 GG	8(17.0)	3(37.5)	
AKT rs1130233 GA	23(48.9)	3(37.5)	0.311
AKT rs1130233 AA	16(34.0)	2(25.0)	0.251
GA+AA vs. GG			0.233
AA vs. GA+GG			0.654
**Male**			
MTOR			
MTOR rs1064261 TT	8(72.7)	5(71.4)	
MTOR rs1064261 TC	3(27.3)	2(28.6)	1.000
MTOR rs1064261 CC	0	0	
Phosphoryltion MTOR			
MTOR rs1064261 TT	21(77.8)	4(50.0)	
MTOR rs1064261 TC	6(22.2)	4(50.0)	0.186
MTOR rs1064261 CC	0	0	
AKT(PAN)			
AKT rs1130233 GG	1(8.3)	1(16.7)	
AKT rs1130233 GA	4(33.3)	4(66.7)	0.282
AKT rs1130233 AA	7(58.3)	1(16.7)	
Phosphoryltion AKT			
AKT rs1130233 GG	5(16.7)	2(40.0)	
AKT rs1130233 GA	15(50.0)	2(40.0)	0.562
AKT rs1130233 AA	10(33.3)	1(20.0)	
**Female**			
MTOR			
MTOR rs1064261 TT	6(100.0)	4(80.0)	
MTOR rs1064261 TC	0	1(20.0)	0.455
MTOR rs1064261 CC	0	0	
Phosphoryltion MTOR			
MTOR rs1064261 TT	9	7	
MTOR rs1064261 TC	0	0	NA
MTOR rs1064261 CC	0	0	
AKT(PAN)			
AKT rs1130233 GG	0	0	
AKT rs1130233 GA	4(80.0)	1(50.0)	
AKT rs1130233 AA	1(20.0)	1(50.0)	1.000
Phosphoryltion AKT			
AKT rs1130233 GG	3(17.6)	1(33.3)	
AKT rs1130233 GA	8(47.1)	1(33.3)	1.000
AKT rs1130233 AA	6(35.3)	1(33.3)	
**H.pylori (+)**			
MTOR			
MTOR rs1064261 TT	2(66.7)	3(75.0)	
MTOR rs1064261 TC	1(33.3)	1(25.0)	1.000
MTOR rs1064261 CC	0	0	
Phosphoryltion MTOR			
MTOR rs1064261 TT	14(82.4)	4(66.7)	
MTOR rs1064261 TC	3(17.6)	2(33.3)	0.576
MTOR rs1064261 CC	0	0	
AKT(PAN)			
AKT rs1130233 GG	0	1(20.0)	
AKT rs1130233 GA	3(42.9)	3(60.0)	0.369
AKT rs1130233 AA	4(57.1)	1(20.0)	
Phosphoryltion AKT			
AKT rs1130233 GG	2(66.7)	2(11.1)	
AKT rs1130233 GA	0	11(61.1)	**0.045**
AKT rs1130233 AA	1(33.3)	5(27.8)	
**H.pylori (-)**			
MTOR			
MTOR rs1064261 TT	7(77.8)	11(84.6)	
MTOR rs1064261 TC	2(22.2)	2(15.4)	1.000
MTOR rs1064261 CC	0	0	
Phosphoryltion MTOR			
MTOR rs1064261 TT	16(84.2)	7(77.8)	
MTOR rs1064261 TC	3(15.8)	2(22.2)	1.000
MTOR rs1064261 CC	0	0	
AKT(PAN)			
AKT rs1130233 GG	1(10.0)	0	
AKT rs1130233 GA	5(50.0)	2(66.7)	1.000
AKT rs1130233 AA	4(40.0)	1(33.3)	
Phosphoryltion AKT			
AKT rs1130233 GG	1(20.0)	6(20.7)	
AKT rs1130233 GA	3(60.0)	12(41.4)	0.826
AKT rs1130233 AA	1(20.0)	11(37.9)	

**Note:**

*using Logistic Regession adjusted by sex, age and *H*.*pylori* infection status.

## Discussion

In this study, we report for the first time an association of the *MTOR* rs1064261 and *AKT* rs1130233 polymorphisms with GC risk and prognosis. In males, the *MTOR* rs1064261 (TC+CC) genotype and the A allele of *AKT* rs1130233 were associated with an increased GC risk. In *H*. *pylori*-negative males, the *AKT* rs1130233 (GA+AA) genotype was associated with an increased AG risk. In addition, the *AKT* rs1130233 (GA+AA) genotype showed a significant interaction with *H*. *pylori* infection in CON→AG→GC progression. The variant genotype of *AKT* rs1130233 was associated with increased p-AKT protein expression. The *AKT* rs1130233 polymorphism was also associated with lymph node metastasis and alcohol drinking. These findings provide experimental evidence to support MTOR and AKT as potential biomarkers of specific types of GC and also provide clues to the interaction between *H*. *pylori* infection and MTOR/AKT signaling.

The *MTOR* gene is located on human chromosome 1p36.2 and encodes the 289 kDa MTOR protein, consisting of 2549 amino acids. MTOR is a member of phosphatidyl inositol kinase (PIK) family and has serine/threonine kinase activities. We found that the *MTOR* gene (TC+CC) variant genotype was associated with an increased GC risk in males, suggesting that it is involved in carcinogenesis. MTOR mainly exists in the cytoplasm under normal conditions and enters the nucleus after activation to regulate the downstream targets eukaryotic initiation factor 4E (eIF-4E) binding protein 1 (4E-BP1) and ribosome 40S small subunit S6 protein kinase (p70S6K) [22–24]. The latter is an essential member of the PI3K/AKT/MTOR pathway and a central metabolic signaling molecule. MTOR integrates a variety of cellular signals from growth factors and nutritional and energy status; thus, it has important biological functions in angiogenesis and cell growth, proliferation, metabolism, migration, differentiation, and apoptosis [25]. Its function is closely linked to the transformation of normal to cancer cells and to cancer cell proliferation. Genetic variations in genes of the MTOR signaling pathway (*PI3K*, *AKT*, and *PTEN*) can promote carcinogenesis [26–28]. *MTOR* SNPs are reported to be associated with susceptibility to GC [7, 9], renal cell carcinoma [6], prostate cancer [8], and esophageal squamous cancer [10]. These SNPs might affect the levels of MTOR expression and transcriptional activity, thereby altering the protein function. The G allele of *MTOR* rs2295080 polymorphism is associated with a reduced GC risk, possibly resulting from reduced promoter activity and mRNA expression [7]. Another *MTOR* polymorphism located in the promoter region, rs1883965 G→A, confers increased risks of GC [9] and esophageal squamous cancer [10]. Our findings suggest that the (TC+CC) genotype is associated with an increased GC risk in males, although no effect on protein expression was observed. *MTOR* can promote cell proliferation and somewhat “oncogenic” characteristics. The variant genotype might have even more oncogenic activity. As men are more likely to be exposed to multiple risk exposure factors (smoking, drinking and unhealthy living habits), carriers of certain genotype may be susceptible to an increased risk of GC.

*AKT*, the v-*AKT* murine thymoma viral oncogene homolog, maps to human chromosome 14q32.32 and encodes a 56 kda protein, consisting of 480 amino acids [15]. AKT is an important effector of the PI3K/AKT/MTOR signal pathway, and genetic mutation or abnormal protein expression can alter a variety of cellular process including migration, proliferation, growth, and survival. Additionally, AKT activation is involved in cell proliferation and apoptosis, which are related to cancer initiation and progression [29]. *AKT* SNPs are reported to be associated with susceptibility to and/or the prognosis of various cancer types including nasopharyngeal carcinoma [12], oral squamous cell carcinoma [13, 30], non-small cell lung cancer [14, 15], pancreatic ductal adenocarcinoma [31], and GC [32] via effects on protein expression and transcriptional activity. Wang et al. studied four SNPs including rs1130233 in a Chinese population [13], and found that three polymorphisms were associated with susceptibility to oral squamous cell carcinoma or DFS, but with no significant relation for rs1130233. Zhang et al. reported that AA haplotypes of *AKT* rs1130233 and rs2494732 conferred an increased nasopharyngeal carcinoma risk [12] and that some *AKT* haplotypes cause increased AKT protein expression [33, 34], leading to altered cellular migration and proliferation. Therefore, the *AKT* rs1130233 A allele might have increased proliferative activity. In this study, we found that the *AKT* rs1130233 A allele conferred an increased risk of GC in males. Generally, males have a higher GC incidence and a higher GC mortality rate than adult women [35]. The high level of exposure of males to environmental risk factors (smoking, drinking and unhealthy living habits) increases their susceptibility to GC [36]. As the A allele might have stronger proliferative activity, the combined effect of *AKT* polymorphism and gender could partially explain the observed high GC risk associated with the *AKT* rs1130233 polymorphism in males.

In addition, we found that in the *H*. *pylori*-negative subgroup, the *AKT* rs1130233 (GA+AA) genotype was associated with increased AG risk. The *AKT* rs1130233 polymorphism showed a significant interaction with *H*. *pylori* infection in CON→AG→GC progression. It is widely accepted that *H*. *pylori* is a major cause of GC. *H*. *pylori* virulent factors may induce abnormal cell proliferation and apoptosis through regulating signaling pathways (including PI3K/AKT); this is a recent research hotspot [37–39]. Tabassam et al. suggested that the AKT phosphorylation induced by the *H*. *pylori* virulence factors cag PAI and OipA regulates intracellular signals responsible for a series of cellular functions involved in gastric carcinogenesis. The specific promotion of AKT serine 473 or threonine 308 phosphorylation by cag PAI and OipA may disrupt downstream proliferation and apoptosis signals. A combination of cag PAI and OipA is sufficient to activate the PI3K/PDK1 pathway and AKT/ERK/ downstream signaling [39]. Nakayama et al. reported that *H*. *pylori* virulence factor VacA induces β-catenin function by activating the PI3K/AKT pathway and inactivating GSK33β [38], thus regulating cell proliferation, differentiation and apoptosis. These data suggest that the interaction of *H*. *pylori* with activated AKT has a biological function. However, neither of the two SNPs included in this study nor *H*. *pylori* infection in the context of these SNPs showed a significant interaction with GC. Overall, these data emphasize the important role of the *AKT* rs1130233 polymorphism in the PI3K/MTOR/AKT pathway. In addition, in the *H*. *pylori*-positive subgroup, those with the *AKT* rs1130233 variant genotype had increased p-AKT expression. As this polymorphism showed an interaction with *H*. *pylori* infection, the effect of this polymorphism on protein expression may only emerge in the presence of *H*. *pylori* infection. A genetic mutation leading to abnormal AKT expression is reported to promote cell migration and proliferation [40, 41], which might explain why this polymorphism increased the risk of AG. Future large-scale studies are required to confirm these results.

We also compared the genotype distribution of these two polymorphisms in groups with different clinicopathological parameters and assessed their relation with GC prognosis. We found that the *AKT* rs1130233 GA, AA, and (GA+AA) genotypes were more frequent in drinkers than in nondrinkers. We therefore assume that drinkers with *AKT* rs1130233 GA, AA, and (GA+AA) genotypes are more susceptible to GC. Avan et al. [31] reported that the *AKT* rs1130233 A allele is associated with reduced survival of pancreatic ductal adenocarcinoma patients, which might be attributed to reduced *AKT1* mRNA and protein expression and reduced apoptosis efficiency [33, 42]. The *AKT* rs1130233 A allele is a risk genotype for cancer, possibly in association with alcohol drinking as consuming alcohol was one of the increased risk factors of GC[43–46]. However, no significant association of these two SNPs with GC survival was found by Cox regression analysis. Due to the relatively small sample size, the recall bias might exist in the case-control study, and outcome status may have caused cases to alter their exposure profile. Therefore, further experiments and large-scale studies were needed to confirm our results.

The present study has some limitations. First, the sample size was relatively small, especially for survival and protein expression analyses, both of which need further confirmation in large populations. Second, only OS was analyzed in the survival analysis and other prognostic parameters such as progression-free survival are also warranted. Third, the survival analysis was only performed in a subset of the whole GC patients which could make the probability of a selective bias, and a large sample size for the prognostic study should be performed in the near future. Fourth, functional experiments are required to elucidate the underlying disease mechanism.

## Conclusion

In summary, the relation of *MTOR* rs1064261 and *AKT* rs1130233 polymorphisms with GC susceptibility and OS has been shown for the first time. *MTOR* rs1064261 and *AKT* rs1130233 polymorphisms were significantly associated with GC risk in males. The *AKT* rs1130233 polymorphism and *H*. *pylori* infection showed a significant interaction in CON→AG→GC progression. The *AKT* rs1130233 polymorphism was associated with increased p-AKT protein expression in *H*. *pylori*-positive individuals. The *AKT* rs1130233 polymorphism was associated with variations in clinicopathological parameters including lymph node metastasis and alcohol drinking. Future large-scale investigations and functional studies are needed to confirm these results.

## Supporting Information

S1 Table ORs (95% CI) of sensitivity analysis for the excluded patients in the survival analysis. (DOC)

S2 Table Association of mTOR rs1064261 and AKT rs1130233 polymorphisms with the risk of atrophic gastritis and gastric cancer stratified by host characteristics. (DOC)

S3 Table Association of mTOR rs1064261 and AKT rs1130233 polymorphisms with the risk of intestinal and diffuse-type gastric cancer. (DOC)

S4 Table Association of mTOR rs1064261 and AKT rs1130233 polymorphisms with the risk of intestinal and diffuse-type gastric cancer stratified by host characteristics. (DOC)

S5 Table Association of the joint effects of mTOR rs1064261 and AKT rs1130233 polymorphisms with the risk of atrophic gastritis and gastric cancer stratified by host characteristics. (DOC)

S6 Table Stratified analysis for the association of mTOR rs1064261 or AKT rs1130233 genptypes with gastric cancer survival. (DOC)

## References

[1] Siegel R, Ward E, Brawley O, Jemal A. Cancer statistics, 2011: the impact of eliminating socioeconomic and racial disparities on premature cancer deaths. CA: a cancer journal for clinicians. 2011;61(4):212–36. 10.3322/caac.20121 . 21685461

[2] Lochhead P, El-Omar EM. Gastric cancer. British medical bulletin. 2008;85:87–100. 10.1093/bmb/ldn007 . 18267927

[3] Tapia O, Riquelme I, Leal P, Sandoval A, Aedo S, Weber H, et al The PI3K/AKT/mTOR pathway is activated in gastric cancer with potential prognostic and predictive significance. Virchows Archiv: an international journal of pathology. 2014;465(1):25–33. Epub 2014/05/23. 10.1007/s00428-014-1588-4 . 24844205

[4] Sanghera KP, Mathalone N, Baigi R, Panov E, Wang D, Zhao X, et al The PI3K/Akt/mTOR pathway mediates retinal progenitor cell survival under hypoxic and superoxide stress. Molecular and cellular neurosciences. 2011;47(2):145–53. 10.1016/j.mcn.2011.03.010 . 21463685

[5] Yu J, Yaba A, Kasiman C, Thomson T, Johnson J. mTOR controls ovarian follicle growth by regulating granulosa cell proliferation. PloS one. 2011;6(7):e21415 10.1371/journal.pone.0021415 21750711 PMC3130037

[6] Cao Q, Ju X, Li P, Meng X, Shao P, Cai H, et al A functional variant in the MTOR promoter modulates its expression and is associated with renal cell cancer risk. PloS one. 2012;7(11):e50302 Epub 2012/12/05. 10.1371/journal.pone.0050302 23209702 PMC3508984

[7] Xu M, Tao G, Kang M, Gao Y, Zhu H, Gong W, et al A polymorphism (rs2295080) in mTOR promoter region and its association with gastric cancer in a Chinese population. PloS one. 2013;8(3):e60080 Epub 2013/04/05. 10.1371/journal.pone.0060080 23555892 PMC3612103

[8] Li Q, Gu C, Zhu Y, Wang M, Yang Y, Wang J, et al Polymorphisms in the mTOR gene and risk of sporadic prostate cancer in an Eastern Chinese population. PloS one. 2013;8(8):e71968 Epub 2013/08/14. 10.1371/journal.pone.0071968 23940798 PMC3734314

[9] He J, Wang MY, Qiu LX, Zhu ML, Shi TY, Zhou XY, et al Genetic variations of mTORC1 genes and risk of gastric cancer in an Eastern Chinese population. Molecular carcinogenesis. 2013;52 Suppl 1:E70–9. Epub 2013/02/21. 10.1002/mc.22013 . 23423739

[10] Zhu ML, Yu H, Shi TY, He J, Wang MY, Li QX, et al Polymorphisms in mTORC1 genes modulate risk of esophageal squamous cell carcinoma in eastern Chinese populations. Journal of thoracic oncology: official publication of the International Association for the Study of Lung Cancer. 2013;8(6):788–95. Epub 2013/03/26. . 23524405 10.1097/JTO.0b013e31828916c6

[11] Shao J, Li Y, Zhao P, Yue X, Jiang J, Liang X, et al Association of mTOR polymorphisms with cancer risk and clinical outcomes: a meta-analysis. PloS one. 2014;9(5):e97085 Epub 2014/05/13. 10.1371/journal.pone.0097085 24816861 PMC4016248

[12] Zhang X, Chen X, Zhai Y, Cui Y, Cao P, Zhang H, et al Combined effects of genetic variants of the PTEN, AKT1, MDM2 and p53 genes on the risk of nasopharyngeal carcinoma. PloS one. 2014;9(3):e92135 Epub 2014/03/19. 10.1371/journal.pone.0092135 24632578 PMC3954877

[13] Wang Y, Lin L, Xu H, Li T, Zhou Y, Dan H, et al Genetic variants in AKT1 gene were associated with risk and survival of OSCC in Chinese Han Population. Journal of oral pathology & medicine: official publication of the International Association of Oral Pathologists and the American Academy of Oral Pathology. 2014 Epub 2014/07/26. 10.1111/jop.12211 . 25060489

[14] Li Q, Yang J, Yu Q, Wu H, Liu B, Xiong H, et al Associations between single-nucleotide polymorphisms in the PI3K-PTEN-AKT-mTOR pathway and increased risk of brain metastasis in patients with non-small cell lung cancer. Clinical cancer research: an official journal of the American Association for Cancer Research. 2013;19(22):6252–60. Epub 2013/10/01. 10.1158/1078-0432.ccr-13-1093 . 24077347

[15] Kim MJ, Kang HG, Lee SY, Jeon HS, Lee WK, Park JY, et al AKT1 polymorphisms and survival of early stage non-small cell lung cancer. Journal of surgical oncology. 2012;105(2):167–74. Epub 2011/08/16. 10.1002/jso.22071 . 21842521

[16] Lauren P. The Two Histological Main Types Of Gastric Carcinoma: Diffuse And So-Called Intestinal-Type Carcinoma. An Attempt at a Histo-Clinical Classification. Acta pathologica et microbiologica Scandinavica. 1965;64:31–49. . 14320675 10.1111/apm.1965.64.1.31

[17] Xu Q, Chen MY, He CY, Sun LP, Yuan Y. Promoter polymorphisms in trefoil factor 2 and trefoil factor 3 genes and susceptibility to gastric cancer and atrophic gastritis among Chinese population. Gene. 2013;529(1):104–12. 10.1016/j.gene.2013.07.070 . 23933418

[18] Dixon MF, Genta RM, Yardley JH, Correa P. Classification and grading of gastritis. The updated Sydney System. International Workshop on the Histopathology of Gastritis, Houston 1994. The American journal of surgical pathology. 1996;20(10):1161–81. . 8827022 10.1097/00000478-199610000-00001

[19] Stolte M, Meining A. The updated Sydney system: classification and grading of gastritis as the basis of diagnosis and treatment. Canadian journal of gastroenterology = Journal canadien de gastroenterologie. 2001;15(9):591–8. . 11573102 10.1155/2001/367832

[20] Xu Q, Yuan Y, Sun LP, Gong YH, Xu Y, Yu XW, et al Risk of gastric cancer is associated with the MUC1 568 A/G polymorphism. International journal of oncology. 2009;35(6):1313–20. . 19885554 10.3892/ijo_00000449

[21] Gong YH, Sun LP, Jin SG, Yuan Y. Comparative study of serology and histology based detection of Helicobacter pylori infections: a large population-based study of 7,241 subjects from China. European journal of clinical microbiology & infectious diseases: official publication of the European Society of Clinical Microbiology. 2010;29(7):907–11. 10.1007/s10096-010-0944-9 . 20440530

[22] Gingras AC, Raught B, Sonenberg N. Regulation of translation initiation by FRAP/mTOR. Genes & development. 2001;15(7):807–26. 10.1101/gad.887201 . 11297505

[23] Browne GJ, Proud CG. Regulation of peptide-chain elongation in mammalian cells. European journal of biochemistry / FEBS. 2002;269(22):5360–8. . 12423334 10.1046/j.1432-1033.2002.03290.x

[24] Martin KA, Blenis J. Coordinate regulation of translation by the PI 3-kinase and mTOR pathways. Advances in cancer research. 2002;86:1–39. . 12374276 10.1016/s0065-230x(02)86001-8

[25] Jiang BH, Liu LZ. PI3K/PTEN signaling in angiogenesis and tumorigenesis. Advances in cancer research. 2009;102:19–65. 10.1016/S0065-230X(09)02002-8 19595306 PMC2933405

[26] Altomare DA, Testa JR. Perturbations of the AKT signaling pathway in human cancer. Oncogene. 2005;24(50):7455–64. 10.1038/sj.onc.1209085 . 16288292

[27] Arcaro A, Guerreiro AS. The phosphoinositide 3-kinase pathway in human cancer: genetic alterations and therapeutic implications. Current genomics. 2007;8(5):271–306. 10.2174/138920207782446160 19384426 PMC2652403

[28] Stiles B, Gilman V, Khanzenzon N, Lesche R, Li A, Qiao R, et al Essential role of AKT-1/protein kinase B alpha in PTEN-controlled tumorigenesis. Molecular and cellular biology. 2002;22(11):3842–51. 11997518 10.1128/MCB.22.11.3842-3851.2002 PMC133830

[29] De Marco C, Rinaldo N, Bruni P, Malzoni C, Zullo F, Fabiani F, et al Multiple genetic alterations within the PI3K pathway are responsible for AKT activation in patients with ovarian carcinoma. PloS one. 2013;8(2):e55362 10.1371/journal.pone.0055362 23408974 PMC3567053

[30] Wang Y, Lin L, Xu H, Li T, Zhou Y, Dan H, et al Genetic variants in AKT1 gene were associated with risk and survival of OSCC in Chinese Han Population. Journal of oral pathology & medicine: official publication of the International Association of Oral Pathologists and the American Academy of Oral Pathology. 2015;44(1):45–50. Epub 2014/07/26. 10.1111/jop.12211 . 25060489

[31] Avan A, Avan A, Le Large TY, Mambrini A, Funel N, Maftouh M, et al AKT1 and SELP polymorphisms predict the risk of developing cachexia in pancreatic cancer patients. PloS one. 2014;9(9):e108057 Epub 2014/09/23. 10.1371/journal.pone.0108057 25238546 PMC4169595

[32] Wang X, Lin Y, Lan F, Yu Y, Ouyang X, Wang X, et al A GG allele of 3'-side AKT1 SNP is associated with decreased AKT1 activation and better prognosis of gastric cancer. Journal of cancer research and clinical oncology. 2014;140(8):1399–411. Epub 2014/04/17. 10.1007/s00432-014-1663-x . 24737346 PMC11824104

[33] Harris SL, Gil G, Robins H, Hu W, Hirshfield K, Bond E, et al Detection of functional single-nucleotide polymorphisms that affect apoptosis. Proceedings of the National Academy of Sciences of the United States of America. 2005;102(45):16297–302. 10.1073/pnas.0508390102 16260726 PMC1283473

[34] Emamian ES, Hall D, Birnbaum MJ, Karayiorgou M, Gogos JA. Convergent evidence for impaired AKT1-GSK3beta signaling in schizophrenia. Nature genetics. 2004;36(2):131–7. 10.1038/ng1296 . 14745448

[35] Jemal A, Bray F, Center MM, Ferlay J, Ward E, Forman D. Global cancer statistics. CA: a cancer journal for clinicians. 2011;61(2):69–90. 10.3322/caac.20107 . 21296855

[36] Brenner H, Rothenbacher D, Arndt V. Epidemiology of stomach cancer. Methods in molecular biology. 2009;472:467–77. 10.1007/978-1-60327-492-0_23 . 19107449

[37] Isomoto H, Moss J, Hirayama T. Pleiotropic actions of Helicobacter pylori vacuolating cytotoxin, VacA. The Tohoku journal of experimental medicine. 2010;220(1):3–14. Epub 2010/01/05. . 20046046 10.1620/tjem.220.3

[38] Nakayama M, Hisatsune J, Yamasaki E, Isomoto H, Kurazono H, Hatakeyama M, et al Helicobacter pylori VacA-induced inhibition of GSK3 through the PI3K/Akt signaling pathway. The Journal of biological chemistry. 2009;284(3):1612–9. Epub 2008/11/11. 10.1074/jbc.M806981200 18996844 PMC2615499

[39] Tabassam FH, Graham DY, Yamaoka Y. Helicobacter pylori activate epidermal growth factor receptor- and phosphatidylinositol 3-OH kinase-dependent Akt and glycogen synthase kinase 3beta phosphorylation. Cellular microbiology. 2009;11(1):70–82. Epub 2008/09/11. 10.1111/j.1462-5822.2008.01237.x 18782353 PMC2827479

[40] Hemmings BA. Akt signaling: linking membrane events to life and death decisions. Science (New York, NY). 1997;275(5300):628–30. . 9019819 10.1126/science.275.5300.628

[41] Downward J. Mechanisms and consequences of activation of protein kinase B/Akt. Current opinion in cell biology. 1998;10(2):262–7. . 9561851 10.1016/s0955-0674(98)80149-x

[42] Giovannetti E, Zucali PA, Peters GJ, Cortesi F, D'Incecco A, Smit EF, et al Association of polymorphisms in AKT1 and EGFR with clinical outcome and toxicity in non-small cell lung cancer patients treated with gefitinib. Molecular cancer therapeutics. 2010;9(3):581–93. 10.1158/1535-7163.MCT-09-0665 . 20159991

[43] Ali Z, Deng Y, Ma C. Progress of research in gastric cancer. Journal of nanoscience and nanotechnology. 2012;12(11):8241–8. . 23421202 10.1166/jnn.2012.6692

[44] Moy KA, Fan Y, Wang R, Gao YT, Yu MC, Yuan JM. Alcohol and tobacco use in relation to gastric cancer: a prospective study of men in Shanghai, China. Cancer epidemiology, biomarkers & prevention: a publication of the American Association for Cancer Research, cosponsored by the American Society of Preventive Oncology. 2010;19(9):2287–97. 10.1158/1055-9965.EPI-10-0362 20699372 PMC2936659

[45] Duell EJ, Travier N, Lujan-Barroso L, Clavel-Chapelon F, Boutron-Ruault MC, Morois S, et al Alcohol consumption and gastric cancer risk in the European Prospective Investigation into Cancer and Nutrition (EPIC) cohort. The American journal of clinical nutrition. 2011;94(5):1266–75. 10.3945/ajcn.111.012351 . 21993435

[46] Tramacere I, Negri E, Pelucchi C, Bagnardi V, Rota M, Scotti L, et al A meta-analysis on alcohol drinking and gastric cancer risk. Annals of oncology: official journal of the European Society for Medical Oncology / ESMO. 2012;23(1):28–36. 10.1093/annonc/mdr135 . 21536659

