# Supplementary material for: Association of MTOR and AKT Gene Polymorphisms with Susceptibility and Survival of Gastric Cancer
Source: PLoS One. 2015 Aug 28;10(8):e0136447. doi: 10.1371/journal.pone.0136447 (PMC4552869; doi:10.1371/journal.pone.0136447)
Supplement: S1 Table — (DOC) [file pone.0136447.s001.doc]

**Table S1**. **ORs (95% CI) of sensitivity analysis.**

| Excluding group | Heterozygote *vs.* wild-type | Mutant type *vs.* wild-type | Dominant model | Recessive model | Mutant allele *vs.* wild-type allele |
| --- | --- | --- | --- | --- | --- |
| one by one | OR (95% CI) | OR (95% CI) | OR (95% CI) | OR (95% CI) | OR (95% CI) |
| MTOR Over all | 0.99(0.75-1.32) | 1.21(0.40-3.60) | 1.00(0.76-1.32) | 1.21(0.41-3.62) | 1.01(0.78-1.31) |
| MTOR rs1064261 Included | 0.92(0.62-1.36) | 1.60(0.45-5.71) | 0.95(0.65-1.39) | 1.62(0.45-5.77) | 0.99(0.70-1.41) |
| MTOR rs1064261 Excluded | 1.09(0.72-1.66) | 0.56(0.07-4.65) | 1.06(0.70-1.60) | 0.55(0.07-4.58) | 1.03(0.70-1.51) |
| AKT Over all | 1.20(0.73-1.97) | 1.30(0.92-1.83) | 1.24(0.80-1.92) | 1.15(0.92-1.44) | 1.13(0.98-1.32) |
| AKT rs1130233 Included | 0.95(0.66-1.37) | 1.12(0.76-1.66) | 1.01(0.72-1.43) | 1.16(0.86-1.57) | 1.07(0.88-1.31) |
| AKT rs1130233 Excluded | 1.58(1.01-2.47) | 1.60(0.99-2.59) | 1.58(1.03-2.43) | 1.14(0.82-1.60) | 1.22(0.97-1.52) |
