# Supplementary material for: Association of MTOR and AKT Gene Polymorphisms with Susceptibility and Survival of Gastric Cancer
Source: PLoS One. 2015 Aug 28;10(8):e0136447. doi: 10.1371/journal.pone.0136447 (PMC4552869; doi:10.1371/journal.pone.0136447)
Supplement: S2 Table — (DOC) [file pone.0136447.s002.doc]

**Supplermentary Table S2. Association of mTOR rs1064261 and AKT rs1130233 polymorphisms with the risk of atrophic gastritis and gastric cancer stratified by host characteristics*.**

| **Variables** | **SNP** | **Gastric mucosa status** | | |  | **AG vs. CON** | |  | **GC vs. AG** | |  | **GC vs. CON** | |  | **GC vs. CON+AG** | |
| --- | --- | --- | --- | --- | --- | --- | --- | --- | --- | --- | --- | --- | --- | --- | --- | --- |
|  |  | **CON(%)** | **AG(%)** | **GC(%)** |  | **OR(95%CI)** | ***P-*value** |  | **OR(95%CI)** | ***P-*value** |  | **OR(95%CI)** | ***P*-value** |  | **OR(95%CI)** | ***P*-value** |
| mTOR rs1064261 | |  |  |  |  |  |  |  |  |  |  |  |  |  |  |  |
| Age |  |  |  |  |  |  |  |  |  |  |  |  |  |  |  |  |
| ≤50 | TT | 223(83.5) | 193(86.2) | 87(83.7) |  | 1(Ref) |  |  | 1(Ref) |  |  | 1(Ref) |  |  | 1(Ref) |  |
|  | TC | 41(15.4) | 29(12.9) | 15(14.4) |  | 0.81(0.46-1.44) | 0.477 |  | 1.14(0.58-2.27) | 0.703 |  | 0.89(0.44-1.80) | 0.737 |  | 1.04(0.56-1.93) | 0.898 |
|  | CC | 3(1.1) | 2(0.9) | 2(1.9) |  | 0.70(0.10-5.05) | 0.720 |  | 2.58(0.35-18.96) | 0.352 |  | 1.14(0.15-8.54) | 0.899 |  | 1.83(0.34-9.96) | 0.483 |
|  | TC+CC vs. TT |  |  |  |  | 0.81(0.46-1.40) | 0.442 |  | 1.23(0.64-2.37) | 0.536 |  | 0.91(0.47-1.79) | 0.794 |  | 1.10(0.61-1.97) | 0.757 |
|  | CC vs. TC+TT |  |  |  |  | 0.73(0.10-5.20) | 0.749 |  | 2.59(0.35-18.98) | 0.350 |  | 1.18(0.16-8.58) | 0.869 |  | 1.82(0.34-9.85) | 0.489 |
|  | C vs.T |  |  |  |  | 0.81(0.48-1.36) | 0.425 |  | 1.30(0.71-2.36) | 0.399 |  | 0.94(0.51-1.74) | 0.850 |  | 1.14(0.67-1.95) | 0.629 |
| >50 | TT | 337(83.0) | 389(84.6) | 299(82.8) |  | 1(Ref) |  |  | 1(Ref) |  |  | 1(Ref) |  |  | 1(Ref) |  |
|  | TC | 66(16.3) | 68(14.8) | 59(16.3) |  | 0.99(0.66-1.47) | 0.945 |  | 1.17(0.79-1.72) | 0.434 |  | 0.95(0.63-1.43) | 0.788 |  | 1.09(0.77-1.53) | 0.637 |
|  | CC | 3(0.7) | 3(0.7) | 3(0.8) |  | 0.58(0.10-3.22) | 0.530 |  | 1.42(0.28-7.19) | 0.670 |  | 1.39(0.26-7.29) | 0.698 |  | 1.27(0.31-5.16) | 0.740 |
|  | TC+CC vs. TT |  |  |  |  | 0.96(0.65-1.43) | 0.854 |  | 1.18(0.81-1.72) | 0.399 |  | 0.96(0.65-1.44) | 0.856 |  | 1.09(0.78-1.53) | 0.602 |
|  | CC vs. TC+TT |  |  |  |  | 0.57(0.10-3.17) | 0.519 |  | 1.39(0.28-7.02) | 0.689 |  | 1.41(0.27-7.49) | 0.685 |  | 1.25(0.31-5.10) | 0.755 |
|  | C vs.T |  |  |  |  | 0.94(0.65-1.36） | 0.759 |  | 1.17(0.82-1.67) | 0.381 |  | 0.99(0.68-1.43) | 0.937 |  | 1.09(0.80-1.49) | 0.579 |
| Sex |  |  |  |  |  |  |  |  |  |  |  |  |  |  |  |  |
| Male | TT | 283(82.7) | 336(87.0) | 260(82.3) |  | 1(Ref) |  |  | 1(Ref) |  |  | 1(Ref) |  |  | 1(Ref) |  |
|  | TC | 55(16.1) | 48(12.4) | 54(17.1) |  | 0.70(0.44-1.12) | 0.138 |  | **1.55(1.00-2.38)** | **0.049** |  | 0.97(0.62-1.51) | 0.880 |  | 1.29(0.89-.186) | 0.184 |
|  | CC | 4(1.2) | 2(0.5) | 2(0.6) |  | 0.28(0.04-1.94) | 0.199 |  | 1.34(0.18-9.79) | 0.775 |  | 0.73(0.12-4.30) | 0.724 |  | 0.87(0.17-0.448) | 0.863 |
|  | TC+CC vs. TT |  |  |  |  | 0.67(0.43-1.06) | 0.087 |  | **1.53(1.00-2.35)** | **0.049** |  | 0.95(0.62-1.47) | 0.824 |  | 1.26(0.88-1.82) | 0.210 |
|  | CC vs. TC+TT |  |  |  |  | 0.31(0.05-2.06) | 0.224 |  | 1.24(0.17-9.01) | 0.835 |  | 0.72(0.12-4.28) | 0.721 |  | 0.82(0.16-4.26) | 0.817 |
|  | C vs.T |  |  |  |  | 0.66(0.43-1.02) | 0.059 |  | 1.47(0.99-2.20) | 0.058 |  | 0.94(0.63-1.41) | 0.772 |  | 1.22(0.87-1.71) | 0.262 |
| Female | TT | 277(83.7) | 248(82.7) | 126(84.6) |  | 1(Ref) |  |  | 1(Ref) |  |  | 1(Ref) |  |  | 1(Ref) |  |
|  | TC | 52(15.7) | 49(16.3) | 20(13.4) |  | 1.19(0.75-1.90) | 0.458 |  | 0.81(0.45-1.44) | 0.471 |  | 0.92(0.50-1.70) | 0.794 |  | 0.85(0.50-1.45) | 0.564 |
|  | CC | 2(0.6) | 3(1.0) | 3(2.0) |  | 1.15(0.17-7.76) | 0.886 |  | 2.30(0.45-11.80) | 0.319 |  | 2.25(0.30-16.91) | 0.430 |  | 2.09(0.46-9.44) | 0.340 |
|  | TC+CC vs. TT |  |  |  |  | 1.19(0.76-1.88) | 0.451 |  | 0.89(0.51-1.54) | 0.673 |  | 0.99(0.55-1.77) | 0.965 |  | 0.92(0.55-1.52) | 0.734 |
|  | CC vs. TC+TT |  |  |  |  | 1.09(0.16-7.42) | 0.930 |  | 2.37(0.46-12.11) | 0.301 |  | 2.32(0.30-17.63) | 0.417 |  | 2.13(0.47-9.63) | 0.326 |
|  | C vs.T |  |  |  |  | 1.17(0.77-1.78) | 0.471 |  | 0.98(0.59-1.62) | 0.930 |  | 1.05(0.61-1.80) | 0.853 |  | 0.99(0.62-1.57) | 0.966 |
| *H.pylori* |  |  |  |  |  |  |  |  |  |  |  |  |  |  |  |  |
| Positive | TT | 124(84.9) | 348(86.4) | 202(81.1) |  | 1(Ref) |  |  | 1(Ref) |  |  | 1(Ref) |  |  | 1(Ref) |  |
|  | TC | 20(13.7) | 51(12.7) | 45(18.1) |  | 0.90(0.51-1.57) | 0.703 |  | 1.56(0.99-2.45) | 0.054 |  | 1.00(0.54-1.87) | 0.997 |  | 1.52(0.99-2.33) | 0.053 |
|  | CC | 2(1.4) | 4(1.0) | 2(0.8) |  | 0.68(0.12-3.84) | 0.662 |  | 0.90(0.16-5.09) | 0.900 |  | 1.19(0.59-2.40) | 0.636 |  | 0.80(0.15-4.14) | 0.789 |
|  | TC+CC vs. TT |  |  |  |  | 0.88(0.51-1.50) | 0.629 |  | 1.51(0.97-2.35) | 0.066 |  | 1.07(0.58-1.97) | 0.827 |  | 1.46(0.97-2.22) | 0.073 |
|  | CC vs. TC+TT |  |  |  |  | 0.67(0.12-3.79) | 0.654 |  | 0.84(0.15-4.81) | 0.849 |  | 1.17(0.72-1.89) | 0.523 |  | 0.75(0.15-3.90) | 0.735 |
|  | C vs.T |  |  |  |  | 0.86(0.53-1.42) | 0.567 |  | 1.41(0.94-2.13) | 0.096 |  | 1.18(0.69-2.02) | 0.537 |  | 1.36(0.93-2.00) | 0.114 |
| Negetive | TT | 432(82.6) | 229(83.9) | 196(85.6) |  | 1(Ref) |  |  | 1(Ref) |  |  | 1(Ref) |  |  | 1(Ref) |  |
|  | TC | 87(16.6) | 43(15.8) | 30(13.1) |  | 0.92(0.62-1.38) | 0.698 |  | 0.89(0.53-1.49) | 0.659 |  | 1.21(0.78-1.87) | 0.400 |  | 0.83(0.53-1.29) | 0.403 |
|  | CC | 4(0.8) | 1(0.4) | 3(1.3) |  | 0.44(0.05-4.08) | 0.472 |  | 5.40(0.53-54.60) | 0.153 |  | 1.29(0.81-2.05) | 0.288 |  | 2.37(0.53-10.59) | 0.258 |
|  | TC+CC vs. TT |  |  |  |  | 0.90(0.61-1.34) | 0.610 |  | 0.98(0.59-1.61) | 0.920 |  | 1.23(0.82-1.86) | 0.316 |  | 0.88(0.58-1.35) | 0.563 |
|  | CC vs. TC+TT |  |  |  |  | 0.45(0.05-4.15) | 0.482 |  | 5.71(0.56-57.80) | 0.140 |  | 1.14(0.80-1.63) | 0.468 |  | 2.45(0.55-10.89) | 0.238 |
|  | C vs.T |  |  |  |  | 0.89(0.61-1.29) | 0.535 |  | 1.07(0.67-1.71) | 0.783 |  | 0.89(0.59-1.35) | 0.581 |  | 0.95(0.64-1.40) | 0.785 |
| AKT rs1130233 |  |  |  |  |  |  |  |  |  |  |  |  |  |  |  |  |
| Age |  |  |  |  |  |  |  |  |  |  |  |  |  |  |  |  |
| ≤50 | GG | 55(20.0) | 45(20.3) | 19(18.3) |  | 1(Ref) |  |  | 1(Ref) |  |  | 1(Ref) |  |  | 1(Ref) |  |
|  | GA | 132(49.4) | 124(55.9) | 54(51.9) |  | 1.21(0.72-2.04) | 0.471 |  | 1.02(0.54-1.92) | 0.952 |  | 1.05(0.55-2.01) | 0.886 |  | 1.08(0.61-1.93) | 0.794 |
|  | AA | 80(30.0) | 53(23.9) | 31(29.8) |  | 0.95(0.53-1.69) | 0.848 |  | 1.42(0.69-2.91) | 0.339 |  | 1.04(0.51-2.12) | 0.910 |  | 1.30(0.69-2.47) | 0.418 |
|  | GA+AA vs. GG |  |  |  |  | 1.11(0.68-1.81) | 0.685 |  | 1.15(0.63-2.11) | 0.655 |  | 1.05(0.56-1.94) | 0.888 |  | 1.16(0.67-2.01) | 0.608 |
|  | AA vs. GA+GG |  |  |  |  | 0.83(0.53-1.29) | 0.407 |  | 1.38(0.81-2.35) | 0.236 |  | 1.02(0.60-1.73) | 0.939 |  | 1.23(0.77-1.98) | 0.388 |
|  | A vs.G |  |  |  |  | 0.96(0.73-1.27) | 0.771 |  | 1.19(0.85-1.67) | 0.321 |  | 1.02(0.72-1.45) | 0.897 |  | 1.14(0.84-1.55) | 0.402 |
| >50 | GG | 89(22.0) | 84(18.5) | 63(17.5) |  | 1(Ref) |  |  | 1(Ref) |  |  | 1(Ref) |  |  | 1(Ref) |  |
|  | GA | 197(48.8) | 237(52.2) | 179(49.6) |  | 1.21(0.83-1.77) | 0.325 |  | 1.05(0.72-1.55) | 0.791 |  | 1.10(0.73-1.65) | 0.642 |  | 1.15(0.81-1.61) | 0.434 |
|  | AA | 118(29.2) | 133(29.3) | 119(33.0) |  | 1.19(0.77-1.84) | 0.435 |  | 1.22(0.80-1.84) | 0.358 |  | 1.27(0.81-1.97) | 0.297 |  | 1.31(0.91-1.90) | 0.147 |
|  | GA+AA vs. GG |  |  |  |  | 1.20(0.84-1.72) | 0.327 |  | 1.12(0.78-1.61) | 0.550 |  | 1.17(0.79-1.71) | 0.437 |  | 1.21(0.88-1.67) | 0.250 |
|  | AA vs. GA+GG |  |  |  |  | 1.02(0.75-1.41) | 0.887 |  | 1.17(0.86-1.57) | 0.322 |  | 1.19(0.86-1.65) | 0.289 |  | 1.18(0.90(1.54) | 0.228 |
|  | A vs.G |  |  |  |  | 1.07(0.87-1.32) | 0.515 |  | 1.11(0.91-1.35) | 0.326 |  | 1.13(0.91-1.41) | 0.256 |  | 1.14(0.95-1.36) | 0.151 |
| Sex |  |  |  |  |  |  |  |  |  |  |  |  |  |  |  |  |
| Male | GG | 74(21.7) | 84(22.0) | 53(16.8) |  | 1(Ref) |  |  | 1(Ref) |  |  | 1(Ref) |  |  | 1(Ref) |  |
|  | GA | 170(49.9) | 200(52.5) | 154(48.7) |  | 0.97(0.64-1.47) | 0.891 |  | 1.32(0.87-2.00) | 0.192 |  | 1.30(0.83-2.05) | 0.255 |  | 1.33(0.91-1.94) | 0.139 |
|  | AA | 97(28.4) | 97(25.5) | 109(34.5) |  | 0.88(0.55-1.42) | 0.609 |  | **1.76(1.12-2.77)** | **0.014** |  | **1.70(1.03-2.81)** | **0.039** |  | **1.71(1.14-2.57)** | **0.009** |
|  | GA+AA vs. GG |  |  |  |  | 0.94(0.64-1.40) | 0.767 |  | 1.46(0.99-2.17) | 0.058 |  | 1.44(0.93-2.23) | 0.099 |  | **1.46(1.02-2.08)** | **0.038** |
|  | AA vs. GA+GG |  |  |  |  | 0.90(0.62-1.30) | 0.565 |  | **1.45(1.04-2.03)** | **0.030** |  | 1.38(0.96-1.98) | 0.083 |  | **1.40(1.04-1.88)** | **0.026** |
|  | A vs.G |  |  |  |  | 0.94(0.75-1.18) | 0.592 |  | **1.32(1.06-1.64)** | **0.013** |  | **1.29(1.01-1.64)** | **0.038** |  | **1.30(1.07-1.58)** | **0.008** |
| Female | GG | 70(21.2) | 45(15.2) | 29(19.5) |  | 1(Ref) |  |  | 1(Ref) |  |  | 1(Ref) |  |  | 1(Ref) |  |
|  | GA | 159(48.2) | 162(54.7) | 79(53.0) |  | 1.57(0.98-2.51) | 0.058 |  | 0.71(0.41-1.25) | 0.233 |  | 0.87(0.49-1.55) | 0.643 |  | 0.86(0.52-1.42) | 0.561 |
|  | AA | 101(30.6) | 89(30.1) | 41(27.5) |  | 1.41(0.84-2.38) | 0.198 |  | 0.66(0.36-1.24) | 0.197 |  | 0.80(0.43-1.48) | 0.478 |  | 0.80(0.46-1.37) | 0.410 |
|  | GA+AA vs. GG |  |  |  |  | 1.49(0.96-2.33) | 0.076 |  | 0.70(0.1-1.20) | 0.192 |  | 0.86(0.50-1.45) | 0.562 |  | 0.84(0.52-1.34) | 0.458 |
|  | AA vs. GA+GG |  |  |  |  | 0.98(0.68-1.42) | 0.929 |  | 0.89(0.57-1.39) | 0.595 |  | 0.85(0.53-1.36) | 0.500 |  | 0.86(0.57-1.30) | 0.467 |
|  | A vs.G |  |  |  |  | 1.12(0.88-1.43) | 0.354 |  | 0.86(0.64-1.15) | 0.298 |  | 0.89(0.66-1.20) | 0.443 |  | 0.89(0.68-1.16) | 0.377 |
| *H.pylori* |  |  |  |  |  |  |  |  |  |  |  |  |  |  |  |  |
| Positive | GG | 24(16.4) | 86(21.6) | 41(16.5) |  | 1(Ref) |  |  | 1(Ref) |  |  | 1(Ref) |  |  | 1(Ref) |  |
|  | GA | 81(55.5) | 205(51.4) | 128(51.4) |  | 0.71(0.42-1.20) | 0.201 |  | 1.46(0.92-2.31) | 0.105 |  | 1.00(0.54-1.87) | 0.997 |  | 1.32(0.85-2.06) | 0.216 |
|  | AA | 41(28.1) | 108(27.1) | 80(32.1) |  | 0.69(0.38-1.24) | 0.214 |  | 1.60(0.97-2.64) | 0.065 |  | 1.19(0.59-2.40) | 0.636 |  | 1.50(0.93-2.44) | 0.098 |
|  | GA+AA vs. GG |  |  |  |  | 0.71(0.43-1.18) | 0.184 |  | 1.50(0.97-2.32) | 0.066 |  | 1.07(0.58-1.97) | 0.827 |  | 1.39(0.91-2.11) | 0.129 |
|  | AA vs. GA+GG |  |  |  |  | 0.94(0.61-1.44) | 0.785 |  | 1.18(0.82-1.70) | 0.363 |  | 0.17(0.72-1.89) | 0.523 |  | 1.18(0.84-1.67) | 0.334 |
|  | A vs.G |  |  |  |  | 0.88(0.67-1.15) | 0.341 |  | 1.22(0.96-1.54) | 0.105 |  | 1.09(0.80-1.49) | 0.595 |  | 1.18(0.95-1.48) | 0.143 |
| Negetive | GG | 118(22.6) | 43(15.9) | 47(20.6) |  | 1(Ref) |  |  | 1(Ref) |  |  | 1(Ref) |  |  | 1(Ref) |  |
|  | GA | 246(47.2) | 149(55.2) | 105(46.1) |  | **1.69(1.12-2.54)** | **0.012** |  | 0.72(0.43-1.19) | 0.194 |  | 1.21(0.78-1.87) | 0.400 |  | 1.02(0.67-1.53) | 0.945 |
|  | AA | 157(30.1) | 78(28.9) | 76(33.3) |  | 1.39(0.89-2.18) | 0.144 |  | 0.94(0.55-1.62) | 0.830 |  | 1.29(0.81-2.05) | 0.288 |  | 1.18(0.76-1.83) | 0.470 |
|  | GA+AA vs. GG |  |  |  |  | **1.57(1.06-2.31)** | **0.024** |  | 0.80(0.50-1.29) | 0.362 |  | 1.23(0.82-1.86) | 0.316 |  | 1.08(0.73-1.58) | 0.716 |
|  | AA vs. GA+GG |  |  |  |  | 0.95(0.68-1.31) | 0.736 |  | 1.21(0.82-1.81) | 0.337 |  | 1.14(0.80-1.63) | 0.468 |  | 1.17(0.84-1.63) | 0.363 |
|  | A vs.G |  |  |  |  | 1.12(0.91-1.39) | 0.280 |  | 1.02(0.78-1.32) | 0.892 |  | 1.14(0.90-1.44) | 0.287 |  | 1.10(0.88-1.36) | 0.421 |

**Note:** *using Logistic Regession adjusted by the other two factors of sex, age and *H.pylori* infection status. When stratified by age, the sex and *H.pylori* infection status were adjusted; when stratified by sex, the age and *H.pylori* infection status were adjusted; and when stratified by *H.pylori* infection status, the sex and age were adjusted.

**Abbreviations:** SNP, single nucleotide polymorphism; CON, control; AG, atrophic gastritis; GC, gastric cancer; OR, odds ratio; CI, confidence interval; Ref, reference.
