# Supplementary material for: Association of MTOR and AKT Gene Polymorphisms with Susceptibility and Survival of Gastric Cancer
Source: PLoS One. 2015 Aug 28;10(8):e0136447. doi: 10.1371/journal.pone.0136447 (PMC4552869; doi:10.1371/journal.pone.0136447)
Supplement: S3 Table — (DOC) [file pone.0136447.s003.doc]

**Supplermentary Table S3. Association of mTOR rs1064261 and AKT rs1130233 polymorphisms with the risk of intestinal and diffuse-type gastric cancer***

| **SNP** | **Gastric mucosa status** | | |  | **Intestinal-type GC vs. CON** | |  | **Diffuse-type GC vs. CON** | |
| --- | --- | --- | --- | --- | --- | --- | --- | --- | --- |
|  | **CON(%)** | **Intestinal-type GC(%)** | **Diffuse-type GC(%)** |  | **OR(95%CI)** | ***P-*value** |  | **OR(95%CI)** | ***P-*value** |
| mTOR rs1064261 |  |  |  |  |  |  |  |  |  |
| TT | 560(83.2) | 120(84.5) | 172(80.8) |  | 1(Ref) |  |  | 1(Ref) |  |
| TC | 107(15.9) | 21(14.8) | 40(18.8) |  | 0.99(0.56-1.74) | 0.963 |  | 1.01(0.68-1.50) | 0.949 |
| CC | 6(0.9) | 1(0.7) | 1(0.5) |  | 0.71(0.08-6.50) | 0.760 |  | 1.42(0.37-5.44) | 0.613 |
| TC+CC vs. TT |  |  |  |  | 0.97(0.56-1.68) | 0.903 |  | 1.04(0.71-1.52) | 0.852 |
| CC vs. TC+TT |  |  |  |  | 0.72(0.80-6.56) | 0.773 |  | 1.41(0.36-5.44) | 0.622 |
| C vs.T |  |  |  |  | 0.95(0.57-1.59) | 0.852 |  | 1.06(0.74-1.50) | 0.766 |
| AKT rs1130233 |  |  |  |  |  |  |  |  |  |
| GG | 144(21.5) | 25(17.6) | 40(18.8) |  | 1(Ref) |  |  | 1(Ref) |  |
| GA | 329(49.0) | 68(47.9) | 115(54.0) |  | 1.19(0.66-2.12) | 0.565 |  | 1.09(0.74-1.62) | 0.667 |
| AA | 198(29.5) | 49(34.5) | 58(27.2) |  | 1.58(0.85-2.91) | 0.147 |  | 1.12(0.73-1.72) | 0.616 |
| GA+AA vs. GG |  |  |  |  | 1.34(0.78-2.30) | 0.297 |  | 1.10(0.75-1.60) | 0.628 |
| AA vs. GA+GG |  |  |  |  | 1.39(0.90-2.15) | 0.140 |  | 1.05(0.77-1.45) | 0.745 |
| A vs.G |  |  |  |  | 1.26(0.94-1.70) | 0.119 |  | 1.05(0.85-1.30) | 0.628 |

**Note:** *using Logistic Regession adjusted by sex, age and *H.pylori* infection status.

**Abbreviations:** SNP, single nucleotide polymorphism; CON, control; GC,gastric cancer; OR, odds ratio; CI, confidence interval; Ref, reference.
