# Supplementary material for: Association of MTOR and AKT Gene Polymorphisms with Susceptibility and Survival of Gastric Cancer
Source: PLoS One. 2015 Aug 28;10(8):e0136447. doi: 10.1371/journal.pone.0136447 (PMC4552869; doi:10.1371/journal.pone.0136447)
Supplement: S4 Table — (DOC) [file pone.0136447.s004.doc]

**Supplermentary Table S4. Association of mTOR rs1064261 and AKT rs1130233 polymorphisms with the risk of intestinal and diffuse-type gastric cancer stratified by host characteristics***

| **Variability** | **SNP** | **Gastric mucosa status** | | |  | **Intestinal-type GC vs. CON** | |  | **Diffuse-type GC vs. CON** | |
| --- | --- | --- | --- | --- | --- | --- | --- | --- | --- | --- |
|  |  | **CON(%)** | **Intestinal-type GC(%)** | **Diffuse-type GC(%)** |  | **OR(95%CI)** | ***P-*value** |  | **OR(95%CI)** | ***P-*value** |
| mTOR rs1064261 |  |  |  |  |  |  |  |  |  |  |
| Age |  |  |  |  |  |  |  |  |  |  |
| ≤50 | TT | 223(83.5) | 17(77.3) | 49(86.0) |  | 1(Ref) |  |  | 1(Ref) |  |
|  | TC | 41(15.4) | 5(22.7) | 8(14.0) |  | 1.45(0.47-4.51) | 0.520 |  | 0.66(0.29-1.51) | 0.322 |
|  | CC | 3(1.1) | 0(0) | 0(0) |  | NA | NA |  | 1.50(0.21-10.95) | 0.688 |
|  | TC+CC vs. TT |  |  |  |  | 1.28(0.42-3.97) | 0.665 |  | 0.73(0.34-1.58) | 0.430 |
|  | CC vs. TC+TT |  |  |  |  | NA | NA |  | 1.59(0.22-11.34) | 0.644 |
|  | C vs.T |  |  |  |  | 1.09(0.39-3.11) | 0.867 |  | 0.82(0.41-1.63) | 0.568 |
| >50 | TT | 337(83.0) | 102(85.7) | 117(78.5) |  | 1(Ref) |  |  | 1(Ref) |  |
|  | TC | 66(16.3) | 16(13.4) | 31(20.8) |  | 0.76(0.40-1.44) | 0.395 |  | 1.18(0.76-1.85) | 0.462 |
|  | CC | 3(0.7) | 1(0.8) | 1(0.7) |  | 1.44(0.14-14.70) | 0.757 |  | 1.39(0.22-8.91) | 0.729 |
|  | TC+CC vs. TT |  |  |  |  | 0.78(0.42-1.6) | 0.422 |  | 1.19(0.77-1.85) | 0.434 |
|  | CC vs. TC+TT |  |  |  |  | 1.52(0.15-15.63) | 0.723 |  | 1.34(0.21-8.74) | 0.757 |
|  | C vs.T |  |  |  |  | 0.83(0.46-1.48) | 0.520 |  | 1.18(0.79-1.77) | 0.427 |
| Sex |  |  |  |  |  |  |  |  |  |  |
| Male | TT | 283(82.7) | 90(84.1) | 100(78.8) |  | 1(Ref) |  |  | 1(Ref) |  |
|  | TC | 55(16.1) | 16(15.0) | 27(21.3) |  | 0.91(0.47-1.78) | 0.781 |  | 1.06(0.65-1.73) | 0.816 |
|  | CC | 4(1.2) | 1(0.9) | 0(0) |  | 1.78(0.18-17.52) | 0.622 |  | 0.46(0.05-4.50) | 0.507 |
|  | TC+CC vs. TT |  |  |  |  | 0.94(0.49-1.81) | 0.861 |  | 1.02(0.63-1.66) | 0.930 |
|  | CC vs. TC+TT |  |  |  |  | 1.70(0.17-16.64) | 0.649 |  | 0.47(0.05-4.55) | 0.514 |
|  | C vs.T |  |  |  |  | 0.98(0.54-1.79) | 0.954 |  | 0.98(0.63-1.54) | 0.944 |
| Female | TT | 277(83.7) | 29(85.3) | 66(83.5) |  | 1(Ref) |  |  | 1(Ref) |  |
|  | TC | 52(15.7) | 5(14.7) | 12(15.2) |  | 1.15(0.40-3.32) | 0.793 |  | 0.93(0.48-1.83) | 0.842 |
|  | CC | 2(0.6) | 0(0) | 1(1.3) |  | NA | NA |  | 3.19(0.44-23.08) | 0.251 |
|  | TC+CC vs. TT |  |  |  |  | 0.98(0.34-2.81) | 0.965 |  | 1.04(0.55-1.97) | 0.897 |
|  | CC vs. TC+TT |  |  |  |  | NA | NA |  | 3.22(0.43-24.10) | 0.255 |
|  | C vs.T |  |  |  |  | 0.83(0.30-2.29) | 0.725 |  | 1.15(0.65-2.03) | 0.644 |
| *H.pylori* |  |  |  |  |  |  |  |  |  |  |
| Positive | TT | 124(84.9) | 66(84.6) | 82(76.6) |  | 1(Ref) |  |  | 1(Ref) |  |
|  | TC | 20(13.7) | 12(15.4) | 24(22.4) |  | 1.38(0.59-3.24) | 0.459 |  | 1.39(0.73-2.65) | 0.313 |
|  | CC | 2(1.4) | 0(0) | 1(0.9) |  | NA | NA |  | 1.12(1.15-8.11) | 0.914 |
|  | TC+CC vs. TT |  |  |  |  | 1.23(0.54-2.84) | 0.625 |  | 1.37(0.74-2.54) | 0.322 |
|  | CC vs. TC+TT |  |  |  |  | NA | NA |  | 1.06(0.15-7.73) | 0.956 |
|  | C vs.T |  |  |  |  | 1.09(0.50-2.35) | 0.835 |  | 1.31(0.74-2.30) | 0.357 |
| Negetive | TT | 432(82.6) | 53(85.5) | 90(84.9) |  | 1(Ref) |  |  | 1(Ref) |  |
|  | TC | 87(16.6) | 8(12.9) | 16(15.1) |  | 0.75(0.33-1.68) | 0.486 |  | 0.83(0.49-1.39) | 0.471 |
|  | CC | 4(0.8) | 1(1.6) | 0(0) |  | 1.60(0.16-15.77) | 0.689 |  | 1.69(0.29-9.95) | 0.560 |
|  | TC+CC vs. TT |  |  |  |  | 0.80(0.37-1.73) | 0.571 |  | 0.86(0.52-1.43) | 0.572 |
|  | CC vs. TC+TT |  |  |  |  | 1.70(1.18-16.43) | 0.648 |  | 1.77(0.30-10.32) | 0.525 |
|  | C vs.T |  |  |  |  | 0.86(0.43-1.75) | 0.685 |  | 0.91(0.57-1.46) | 0.707 |
| AKT rs1130233 |  |  |  |  |  |  |  |  |  |  |
| Age |  |  |  |  |  |  |  |  |  |  |
| ≤50 | GG | 55(20.0) | 4(18.2) | 9(15.8) |  | 1(Ref) |  |  | 1(Ref) |  |
|  | GA | 132(49.4) | 12(54.5) | 32(56.1) |  | 1.13(0.33-3.96) | 0.844 |  | 1.02(0.51-2.06) | 0.959 |
|  | AA | 80(30.0) | 6(27.3) | 16(28.1) |  | 1.06(0.27-4.12) | 0.937 |  | 0.95(0.44-2.06) | 0.893 |
|  | GA+AA vs. GG |  |  |  |  | 1.11(0.35-3.59) | 0.858 |  | 0.99(0.51-1.92) | 0.966 |
|  | AA vs. GA+GG |  |  |  |  | 1.03(0.37-2.84) | 0.961 |  | 0.94(0.53-1.69) | 0.845 |
|  | A vs.G |  |  |  |  | 1.05(0.54-2.01) | 0.893 |  | 0.97(0.67-1.42) | 0.880 |
| >50 | GG | 89(22.0) | 21(17.6) | 27(18.1) |  | 1(Ref) |  |  | 1(Ref) |  |
|  | GA | 197(48.8) | 55(46.2) | 82(55.0) |  | 1.09(0.59-2.00) | 0.786 |  | 1.06(-0.67-1.69) | 0.804 |
|  | AA | 118(29.2) | 43(36.1) | 40(26.8) |  | 1.48(0.77-2.84) | 0.245 |  | 1.17(0.71-1.94) | 0.533 |
|  | GA+AA vs. GG |  |  |  |  | 1.24(0.70-2.19) | 0.471 |  | 1.11(0.72-1.72) | 0.648 |
|  | AA vs. GA+GG |  |  |  |  | 1.43(0.90-2.28) | 0.131 |  | 1.12(0.77-1.62) | 0.555 |
|  | A vs.G |  |  |  |  | 1.25(0.91-1.72) | 0.161 |  | 1.08(0.85-1.38) | 0.522 |
| Sex |  |  |  |  |  |  |  |  |  |  |
| Male | GG | 74(21.7) | 19(17.8) | 18(14.2) |  | 1(Ref) |  |  | 1(Ref) |  |
|  | GA | 170(49.9) | 51(47.7) | 66(52.0) |  | 1.37(0.69-2.72) | 0.374 |  | 1.27(0.76-2.12) | 0.364 |
|  | AA | 97(28.4) | 37(34.6) | 43(33.9) |  | 2.00(0.94-4.23) | 0.071 |  | 1.65(0.94-2.91) | 0.084 |
|  | GA+AA vs. GG |  |  |  |  | 1.57(0.82-3.01) | 0.175 |  | 1.41(0.86-2.30) | 0.175 |
|  | AA vs. GA+GG |  |  |  |  | 1.59(0.94-2.68) | 0.085 |  | 1.35(0.90-2.020 | 0.148 |
|  | A vs.G |  |  |  |  | 1.41(0.99-2.01) | 0.058 |  | 1.27(0.97-1.66) | 0.086 |
| Female | GG | 70(21.2) | 6(17.6) | 18(22.8) |  | 1(Ref) |  |  | 1(Ref) |  |
|  | GA | 159(48.2) | 16(47.1) | 48(60.8) |  | 0.79(0.27-2.32) | 0.674 |  | 0.81(0.44-1.52) | 0.516 |
|  | AA | 101(30.6) | 12(35.3) | 13(16.5) |  | 0.96(0.32-2.91) | 0.939 |  | 0.64(0.32-1.27) | 0.202 |
|  | GA+AA vs. GG |  |  |  |  | 0.91(0.34-2.42) | 0.844 |  | 0.75(0.42-1.34) | 0.332 |
|  | AA vs. GA+GG |  |  |  |  | 1.02(0.46-2.29) | 0.959 |  | 0.70(0.41-1.20) | 0.195 |
|  | A vs.G |  |  |  |  | 0.98(0.57-1.69) | 0.943 |  | 0.79(0.56-1.10) | 0.167 |
| *H.pylori* |  |  |  |  |  |  |  |  |  |  |
| Positive | GG | 24(16.4) | 15(19.2) | 16(15.0) |  | 1(Ref) |  |  | 1(Ref) |  |
|  | GA | 81(55.5) | 37(47.4) | 60(56.1) |  | 0.69(0.30-1.57) | 0.377 |  | 1.14(0.57-2.28) | 0.704 |
|  | AA | 41(28.1) | 26(33.3) | 31(29.0) |  | 0.93(0.35-2.46) | 0.883 |  | 1.30(0.60-2.83) | 0.513 |
|  | GA+AA vs. GG |  |  |  |  | 0.77(0.34-1.76) | 0.537 |  | 1.21(0.62-2.36) | 0.585 |
|  | AA vs. GA+GG |  |  |  |  | 1.22(0.63-2.36) | 0.563 |  | 1.12(0.66-1.89) | 0.682 |
|  | A vs.G |  |  |  |  | 1.01(0.65-1.57) | 0.956 |  | 1.10(0.78-1.55) | 0.587 |
| Negetive | GG | 118(22.6) | 9(14.5) | 24(22.6) |  | 1(Ref) |  |  | 1(Ref) |  |
|  | GA | 246(47.2) | 30(48.4) | 55(51.9) |  | 2.01(0.84-4.80) | 0.115 |  | 1.06(0.65-1.71) | 0.828 |
|  | AA | 157(30.1) | 23(37.1) | 27(25.5) |  | 2.37(1.00-5.62) | 0.050 |  | 1.06(0.63-1.78) | 0.829 |
|  | GA+AA vs. GG |  |  |  |  | 2.12(0.95-.73) | 0.066 |  | 1.05(0.67-1.65) | 0.823 |
|  | AA vs. GA+GG |  |  |  |  | 1.54(0.86-2.74) | 0.146 |  | 1.02(0.68-1.53) | 0.921 |
|  | A vs.G |  |  |  |  | **1.53(1.02-2.29)** | **0.041** |  | 1.03(0.79-1.34) | 0.843 |

**Note:** *using Logistic Regession adjusted by the other two factors of sex, age and *H.pylori* infection status.

**Abbreviations:** SNP, single nucleotide polymorphism; CON, control; GC, gastric cancer; OR, odds ratio; CI, confidence interval; Ref, reference; NA, not available.
