# Supplementary material for: Association of MTOR and AKT Gene Polymorphisms with Susceptibility and Survival of Gastric Cancer
Source: PLoS One. 2015 Aug 28;10(8):e0136447. doi: 10.1371/journal.pone.0136447 (PMC4552869; doi:10.1371/journal.pone.0136447)
Supplement: S5 Table — (DOC) [file pone.0136447.s005.doc]

**Supplermentary Table S5. Association of the joint effects of mTOR rs1064261 and AKT rs1130233 polymorphisms with the risk of atrophic gastritis and gastric cancer stratified by host characteristics***

| **Stratified** | **SNP** | **SNP** | **Gastric mucosa status** | | |  | **AG vs. CON** | |  | **GC vs. AG** | |  | **GC vs. CON** | |  | **GC vs. CON+AG** | |
| --- | --- | --- | --- | --- | --- | --- | --- | --- | --- | --- | --- | --- | --- | --- | --- | --- | --- |
|  |  |  | **CON(%)** | **AG(%)** | **GC(%)** |  | **OR(95%CI)** | ***P-*value** |  | **OR(95%CI)** | ***P-*value** |  | **OR(95%CI)** | ***P*-value** |  | **OR(95%CI)** | ***P*-value** |
| Total | mTOR rs1064261 | AKT rs1130233 |  |  |  |  |  |  |  |  |  |  |  |  |  |  |  |
|  | TT | GG | 114(17.0) | 109(16.2) | 74(15.4) |  | 1(Ref) |  |  | 1(Ref) |  |  | 1(Ref) |  |  | 1(Ref) |  |
|  | TT | GA+AA | 445(66.3) | 463(68.8) | 327(67.8) |  | 1.12(0.82-1.55) | 0.478 |  | 1.13(0.80-1.60) | 0.494 |  | 1.09(0.75-1.58) | 0.648 |  | 1.18(0.86-1.61) | 0.309 |
|  | TC+CC | GG | 30(4.5) | 19(2.8) | 15(3.1) |  | 0.79(0.40-1.58) | 0.510 |  | 1.20(.054-2.64) | 0.658 |  | 0.70(0.32-1.55) | 0.376 |  | 0.96(0.48-1.93) | 0.914 |
|  | TC+CC | GA+AA | 82(12.2) | 82(12.2) | 66(13.7) |  | 1.06(0.68-1.66) | 0.789 |  | 1.40(0.89-2.22) | 0.147 |  | 1.17(0.72-1.92) | 0.526 |  | 1.38(0.92-2.08) | 0.125 |
| Age |  |  |  |  |  |  |  |  |  |  |  |  |  |  |  |  |  |
| ≤50 | TT | GG | 41(15.4) | 36(16.3) | 17(16.3) |  | 1(Ref) |  |  | 1(Ref) |  |  | 1(Ref) |  |  | 1(Ref) |  |
|  | TT | GA+AA | 182(68.2) | 154(69.7) | 70(67.3) |  | 1.17(0.67-2.04) | 0.582 |  | 0.99(0.51-1.90) | 0.964 |  | 0.87(0.45-1.71) | 0.691 |  | 0.97(0.54-1.77) | 0.929 |
|  | TC+CC | GG | 14(5.2) | 9(4.1) | 2(1.9) |  | 1.10(0.39-3.13) | 0.853 |  | 0.50(0.10-2.63) | 0.416 |  | 0.37(0.07-1.99) | 0.247 |  | 0.44(0.09-2.09) | 0.304 |
|  | TC+CC | GA+AA | 30(11.2) | 22(10.0) | 15(14.4) |  | 0.86(0.39-1.91) | 0.717 |  | 1.47(0.60-3.58) | 0.399 |  | 1.00(0.40-2.49) | 0.996 |  | 1.32(0.60-2.92) | 0.493 |
| >50 | TT | GG | 73(48.1) | 73(16.2) | 51(14.1) |  | 1(Ref) |  |  | 1(Ref) |  |  | 1(Ref) |  |  | 1(Ref) |  |
|  | TT | GA+AA | 263(65.1) | 308(68.3) | 248(68.7) |  | 1.12(0.76-1.66) | 0.565 |  | 1.18(0.79-1.75) | 0.427 |  | 1.14(0.74-1.74) | 0.556 |  | 1.24(0.87-1.77) | 0.240 |
|  | TC+CC | GG | 16(4.0) | 10(2.2) | 12(3.3) |  | 0.64(0.25-1.59) | 0.333 |  | 1.64(0.64-4.17) | 0.302 |  | 0.84(0.36-2.15) | 0.770 |  | 1.24(0.57-2.71) | 0.588 |
|  | TC+CC | GA+AA | 52(12.9) | 60(13.3) | 50(13.9) |  | 1.20(0.70-2.04) | 0.511 |  | 1.30(0.77-2.19) | 0.333 |  | 1.14(0.65-2.01) | 0.642 |  | 1.32(0.83-2.11) | 0.241 |
| Sex |  |  |  |  |  |  |  |  |  |  |  |  |  |  |  |  |  |
| Male | TT | GG | 60(17.6) | 74(19.5) | 44(13.9) |  | 1(Ref) |  |  | 1(Ref) |  |  | 1(Ref) |  |  | 1(Ref) |  |
|  | TT | GA+AA | 223(65.4) | 256(67.5) | 216(68.4) |  | 0.90(0.59-1.38) | 0.633 |  | 1.43(0.94-2.19) | 0.097 |  | 1.33(0.83-2.15) | 0.236 |  | 1.40(0.95-2.07) | 0.088 |
|  | TC+CC | GG | 14(4.1) | 10(2.6) | 9(2.8) |  | 0.56(0.21-1.48) | 0.242 |  | 1.37(0.49-3.80) | 0.545 |  | 0.68(0.24-1.92) | 0.466 |  | 1.02(0.42-2.49) | 0.959 |
|  | TC+CC | GA+AA | 44(12.9) | 39(10.3) | 47(14.9) |  | 0.63(0.34-1.17) | 0.145 |  | **2.25(1.26-4.01)** | **0.006** |  | 1.40(0.75-2.60) | 0.288 |  | **1.87(1.12-3.10)** | **0.016** |
| Female | TT | GG | 54(16.4) | 35(11.9) | 24(16.1) |  | 1(Ref) |  |  | 1(Ref) |  |  | 1(Ref) |  |  | 1(Ref) |  |
|  | TT | GA+AA | 222(67.3) | 207(70.4) | 102(68.5) |  | 1.54(0.93-2.54) | 0.090 |  | 0.68(0.38-1.24) | 0.210 |  | 0.79(0.44-1.42) | 0.426 |  | 0.82(0.49-1.37) | 0.444 |
|  | TC+CC | GG | 16(4.8) | 9(3.1) | 5(3.4) |  | 1.21(0.45-3.23) | 0.708 |  | 0.87(0.25-3.04) | 0.831 |  | 0.71(0.21-2.32) | 0.566 |  | 0.84(0.28-2.50) | 0.754 |
|  | TC+CC | GA+AA | 38(11.5) | 43(14.6) | 18(12.1) |  | 1.94(1.00-3.75) | 0.050 |  | 0.59(0.27-1.28) | 0.180 |  | 0.85(0.38-1.92) | 0.698 |  | 0.74(0.37-1.51) | 0.409 |
| *H.pylori* |  |  |  |  |  |  |  |  |  |  |  |  |  |  |  |  |  |
| Positive | TT | GG | 20(13.7) | 75(18.8) | 32(12.9) |  | 1(Ref) |  |  | 1(Ref) |  |  | 1(Ref) |  |  | 1(Ref) |  |
|  | TT | GA+AA | 104(71.2) | 268(67.3) | 170(68.3) |  | 0.68(0.40-1.18) | 0.172 |  | 1.62(1.00-2.62) | 0.051 |  | 1.09(0.56-2.15) | 0.800 |  | 1.46(0.92-2.34) | 0.112 |
|  | TC+CC | GG | 4(2.7) | 11(2.8) | 9(3.6) |  | 0.71(0.20-2.50) | 0.599 |  | 2.07(0.73-5.84) | 0.169 |  | 1.34(0.32-5.58) | 0.683 |  | 1.82(0.68-4.87) | 0.237 |
|  | TC+CC | GA+AA | 18(12.3) | 44(11.1) | 38(15.3) |  | 0.64(0.30-1.34) | 0.234 |  | **2.23(1.19-4.17)** | **0.012** |  | 1.36(0.58-3.17) | 0.482 |  | **2.01(1.11-3.66)** | **0.022** |
| Negetive | TT | GG | 92(17.7) | 34(12.7) | 42(18.4) |  | 1(Ref) |  |  | 1(Ref) |  |  | 1(Ref) |  |  | 1(Ref) |  |
|  | TT | GA+AA | 339(65.1) | 190(71.2) | 153(67.1) |  | **1.57(1.01-2.43)** | **0.043** |  | 0.72(0.43-1.22) | 0.219 |  | 1.10(0.70-1.72) | 0.678 |  | 0.97(0.64-1.48) | 0.901 |
|  | TC+CC | GG | 26(5.0) | 8(3.0) | 5(2.2) |  | 0.89(0.37-2.18) | 0.801 |  | 0.59(1.17-2.04) | 0.405 |  | 0.48(0.17-1.40) | 0.180 |  | 0.54(0.19-1.52) | 0.243 |
|  | TC+CC | GA+AA | 64(12.3) | 35(13.1) | 28(12.3) |  | 1.47(0.82-2.61) | 0.193 |  | 0.79(0.39-1.58) | 0.500 |  | 1.08(0.58-1.98) | 0.812 |  | 0.98(0.55-1.74) | 0.943 |

**Note:** *using Logistic Regession adjusted by the other two factors of sex, age and *H.pylori* infection status.

**Abbreviations:** SNP, single nucleotide polymorphism; CON, control; AG, atrophic gastritis; GC, gastric cancer; OR, odds ratio; CI, confidence interval; Ref, reference.
