# Supplementary material for: Association of MTOR and AKT Gene Polymorphisms with Susceptibility and Survival of Gastric Cancer
Source: PLoS One. 2015 Aug 28;10(8):e0136447. doi: 10.1371/journal.pone.0136447 (PMC4552869; doi:10.1371/journal.pone.0136447)
Supplement: S6 Table — (DOC) [file pone.0136447.s006.doc]

**Supplermentary Table S6. Stratified analysis for the association of mTOR rs1064261 or AKT rs1130233 genptypes with gastric cancer survival**

| **Variables** | **Death** | **Heterozygous vs. Wild** | |  | **Mutation vs. Wild** | |  | **Dominance model** | |  | **Recessive model** | |
| --- | --- | --- | --- | --- | --- | --- | --- | --- | --- | --- | --- | --- |
|  | **/patients** | ***P*** | **HR(95%CI)** |  | ***P*** | **HR(95%CI)** |  | ***P*** | **HR(95%CI)** |  | ***P*** | **HR(95%CI)** |
| mTOR rs1064261 |  |  |  |  |  |  |  |  |  |  |  |  |
| Age |  |  |  |  |  |  |  |  |  |  |  |  |
| ≤50 | 20/54 | 0.666 | 0.76(0.22-2.61) |  | NA | NA |  | 0.666 | 0.76(0.22-2.61) |  | NA | NA |
| >50 | 47/151 | 0.715 | 0.86(0.39-1.92) |  | 0.666 | 0.05(0-44423.96) |  | 0.628 | 0.82(0.37-1.83) |  | 0.673 | 0.05(0-58051.02) |
| Sex |  |  |  |  |  |  |  |  |  |  |  |  |
| Male | 42/142 | 0.949 | 0.98(0.45-2.11) |  | NA | NA |  | 0.949 | 0.98(0.45-2.11) |  | NA | NA |
| Female | 25/63 | 0.500 | 0.61(0.14-2.59) |  | 0.628 | 0.05(0-10311.10) |  | 0.381 | 0.52(0.12-2.23) |  | 0.638 | 0.05(0-14727.33) |
| Borrmann type |  |  |  |  |  |  |  |  |  |  |  |  |
| Borrmann Ⅰ-Ⅱ | 29/76 | 0.903 | 1.06(0.41-2.78) |  | 0.637 | 0.05(0-14015.93) |  | 0.935 | 0.96(0.37-2.52) |  | 0.638 | 0.05(0-14532.20) |
| Borrmann Ⅲ-Ⅳ | 37/101 | 0.309 | 0.58(0.21-1.65) |  | NA | NA |  | 0.309 | 0.58(0.21-1.65) |  | NA | NA |
| Lauren grade |  |  |  |  |  |  |  |  |  |  |  |  |
| Intestinal | 18/75 | 0.614 | 0.69(0.16-2.98) |  | NA | NA |  | 0.614 | 0.69(0.16-2.98) |  | NA | NA |
| Diffuse | 49/129 | 0.630 | 0.83(0.39-1.77) |  | 0.631 | 0.05(0-11124.50) |  | 0.538 | 0.79(0.37-1.68) |  | 0.638 | 0.05(0-1408.02) |
| TNM stage |  |  |  |  |  |  |  |  |  |  |  |  |
| Ⅰ-Ⅱ | 22/120 | 0.913 | 1.06(0.36-3.14) |  | 0.754 | NA |  | 0.994 | 1.00(0.34-2.97) |  | 0.754 | NA |
| Ⅲ-Ⅳ | 45/85 | 0.424 | 0.70(0.30-1.67) |  | NA | NA |  | 0.424 | 0.70(0.30-1.67) |  | NA | NA |
| Growth pattern |  |  |  |  |  |  |  |  |  |  |  |  |
| Massive and Nested | 12/70 | 0.430 | 0.44(0.06-3.40) |  | NA | NA |  | 0.430 | 0.44(0.06-3.40) |  | NA | NA |
| Diffused | 24/64 | 0.425 | 0.61(0.18-2.05) |  | NA | NA |  | 0.425 | 0.61(0.18-2.05) |  | NA | NA |
| Depth of invasion |  |  |  |  |  |  |  |  |  |  |  |  |
| T1+T2 | 1/43 | 0.780 | 0.04(0-3.73×10-8) |  | NA | NA |  | 0.780 | 0.04(0-3.73×10-8) |  | NA | NA |
| T3+T4 | 35/91 | 0.283 | 0.57(0.20-1.60) |  | NA | NA |  | 0.283 | 0.57(0.20-1.60) |  | NA | NA |
| Lymphatic metastasis |  |  |  |  |  |  |  |  |  |  |  |  |
| Negative | 11/77 | 0.805 | 0.82(0.18-3.82) |  | 0.776 | 0.05(0-5.90×10-7) |  | 0.735 | 0.77(0.17-3.55) |  | 0.781 | 0.05(0-8.72×10-7) |
| Positive | 56/128 | 0.928 | 0.97(0.46-2.04) |  | NA | NA |  | 0.928 | 0.97(0.46-2.04) |  | NA | NA |
| Smoking |  |  |  |  |  |  |  |  |  |  |  |  |
| Never Smoker | 21/80 | 0.825 | 0.87(0.26-2.96) |  | NA | NA |  | 0.825 | 0.87(0.26-2.96) |  | NA | NA |
| Ever Smoker | 15/54 | 0.210 | 0.27(0.04-2.08) |  | NA | NA |  | 0.210 | 0.27(0.04-2.08) |  | NA | NA |
| Alcohol drinking |  |  |  |  |  |  |  |  |  |  |  |  |
| Nondrinker | 24/90 | 0.838 | 0.88(0.26-2.96) |  | NA | NA |  | 0.838 | 0.88(0.26-2.96) |  | NA | NA |
| Drinker | 12/44 | 0.176 | 0.24(0.03-1.89) |  | NA | NA |  | 0.176 | 0.24(0.03-1.89) |  | NA | NA |
| Family history |  |  |  |  |  |  |  |  |  |  |  |  |
| No | 33/107 | 0.214 | 0.47(0.14-1.54) |  | NA | NA |  | 0.214 | 0.47(0.14-1.54) |  | NA | NA |
| Yes | 3/27 | 0.490 | 2.33(0.21-25.75) |  | NA | NA |  | 0.490 | 2.33(0.21-25.75) |  | NA | NA |
| *H. pylor*i-IgG |  |  |  |  |  |  |  |  |  |  |  |  |
| Negative | 29/86 | 0.742 | 1.20(0.41-3.45) |  | 0.658 | 0.05(0-32008.06) |  | 0.920 | 1.06(0.37-3.05) |  | 0.655 | 0.05(0-28749.51) |
| Positive | 37/117 | 0.445 | 0.71(0.30-1.71) |  | NA | NA |  | 0.445 | 0.71(0.30-1.71) |  | NA | NA |
| AKT rs1130233 |  |  |  |  |  |  |  |  |  |  |  |  |
| Age |  |  |  |  |  |  |  |  |  |  |  |  |
| ≤50 | 20/54 | 0.890 | 1.09(0.31-3.88) |  | 0.707 | 0.76(0.18-3.20) |  | 0.949 | 0.96(0.28-3.28) |  | 0.575 | 0.75(0.27-2.06) |
| >50 | 47/151 | 0.775 | 0.88(0.38-2.08) |  | 0.648 | 1.22(0.51-2.91) |  | 0.987 | 1.01(4.51-2.25) |  | 0.274 | 1.39(0.77-2.38) |
| Sex |  |  |  |  |  |  |  |  |  |  |  |  |
| Male | 42/142 | 0.823 | 1.12(0.42-3.00) |  | 0.415 | 1.51(0.56-4.07) |  | 0.623 | 1.26(0.50-3.22) |  | 0.271 | 1.41(0.77-2.60) |
| Female | 25/63 | 0.624 | 0.78(0.28-2.15) |  | 0.716 | 0.80(0.24-2.65) |  | 0.670 | 0.81(0.30-2.17) |  | 0.807 | 0.89(0.36-2.23) |
| Borrmann type |  |  |  |  |  |  |  |  |  |  |  |  |
| Borrmann Ⅰ-Ⅱ | 29/76 | 0.169 | 0.52(0.21-1.32) |  | 0.148 | 0.48(0.18-1.30) |  | 0.113 | 0.50(0.21-1.18) |  | 0.633 | 0.83(0.38-1.81) |
| Borrmann Ⅲ-Ⅳ | 37/101 | 0.286 | 1.94(0.57-6.56) |  | 0.146 | 2.51(0.72-8.73) |  | 0.186 | 2.22(0.68-7.25) |  | 0.332 | 1.38(0.72-2.67) |
| Lauren grade |  |  |  |  |  |  |  |  |  |  |  |  |
| Intestinal | 18/75 | 0.984 | 1.02(0.21-4.90) |  | 0.476 | 1.75(0.38-8.11) |  | 0.713 | 1.32(0.30-5.74) |  | 0.239 | 1.74(0.69-4.40) |
| Diffuse | 49/129 | 0.912 | 0.96(0.43-2.11) |  | 0.950 | 1.03(0.44-2.43) |  | 0.952 | 0.98(0.6-2.09) |  | 0.863 | 1.06(0.57-1.94) |
| TNM stage |  |  |  |  |  |  |  |  |  |  |  |  |
| Ⅰ-Ⅱ | 22/120 | 0.330 | 0.59(0.21-1.70) |  | 0.364 | 0.58(0.18-1.89) |  | 0.286 | 0.58(0.21-1.58) |  | 0.769 | 0.87(0.34-2.22) |
| Ⅲ-Ⅳ | 45/85 | 0.434 | 1.48(0.56-3.90) |  | 0.360 | 1.59(0.59-4.31) |  | 0.398 | 1.49(0.59-3.79) |  | 0.605 | 1.17(0.64-2.13) |
| Growth pattern |  |  |  |  |  |  |  |  |  |  |  |  |
| Massive and Nested | 12/70 | 0.704 | 1.51(0.18-12.54) |  | 0.618 | 1.73(0.20-14,85) |  | 0.662 | 1.58(0.20-12.24) |  | 0.651 | 1.30(0.41-4.11) |
| Diffused | 24/64 | 0.972 | 1.02(0.29-3.63) |  | 0.566 | 1.47(0.40-5.43) |  | 0.796 | 1.17(0.35-3.93) |  | 0.409 | 1.42(0.62-3.24) |
| Depth of invasion |  |  |  |  |  |  |  |  |  |  |  |  |
| T1+T2 | 1/43 | 0.678 | 0(0-2.23×10-11) |  | 0.584 | 0.01(0-852702.70) |  | 0.817 | 0(0-4.83×10-29) |  | 0.697 | 0.03(0-1.79×10-6) |
| T3+T4 | 35/91 | 0.455 | 1.59(0.47-5.42) |  | 0.316 | 1.90(0.54-6.61) |  | 0.371 | 1.72(0.53-5.61) |  | 0.493 | 1.27(0.64-2.49) |
| Lymphatic metastasis |  |  |  |  |  |  |  |  |  |  |  |  |
| Negative | 11/77 | 0.834 | 1.25(0.16-10.07) |  | 0.742 | 0.67(0.06-7.46) |  | 0.975 | 1.03(0.13-8.11) |  | 0.438 | 0.55(0.12-2.52) |
| Positive | 56/128 | 0.578 | 1.24(0.58-2.66) |  | 0.416 | 1.38(0.63-3.02) |  | 0.514 | 1.27(0.62-2.59) |  | 0.483 | 1.21(0.71-2.08) |
| Smoking |  |  |  |  |  |  |  |  |  |  |  |  |
| Never Smoker | 21/80 | 0.365 | 0.58(0.18-1.89) |  | 0.822 | 0.87(0.26-2.90) |  | 0.490 | 0.68(0.23-2.03) |  | 0.510 | 1.35(0.56-3.25) |
| Ever Smoker | 15/54 | 0.367 | 27.74(0.02-37843.86) |  | 0.364 | 34.03(0.02-69030.70) |  | 0.341 | 25.00(0.03-18798.85) |  | 0.684 | 1.24(0.44-3.48) |
| Alcohol drinking |  |  |  |  |  |  |  |  |  |  |  |  |
| Nondrinker | 24/90 | 0.962 | 1.03(0.33-3.19) |  | 0.862 | 1.11(0.34-3.70) |  | 0.920 | 1.06(0.36-3.09) |  | 0.812 | 1.11(0.47-2.60) |
| Drinker | 12/44 | 0.634 | 22.88(0-9.07×10-6) |  | 0.586 | 24.73(0-2.54×10-6) |  | 0.608 | 21.81(0-2.80×10-6) |  | 0.356 | 1.71(0.55-5.30) |
| Family history |  |  |  |  |  |  |  |  |  |  |  |  |
| No | 33/107 | 0.680 | 0.80(0.27-2.36) |  | 0.953 | 1.04(0.33-3.21) |  | 0.807 | 0.88(0.31-2.50) |  | 0.527 | 1.26(0.62-2.56) |
| Yes | 3/27 | 0.661 | 42.98(0-8.67×10-8) |  | 0.542 | 41.72(0-×10-6) |  | 0.563 | 30.11(0-3.06×10-6) |  | 0.379 | 2.94(0.27-32.42) |
| *H. pylor*i-IgG |  |  |  |  |  |  |  |  |  |  |  |  |
| Negative | 29/86 | 0.398 | 0.66(0.25-1.72) |  | 0.474 | 0.69(0.24-1.93) |  | 0.379 | 0.67(0.27-1.64) |  | 0.926 | 0.96(0.44-2.12) |
| Positive | 37/117 | 0.617 | 1.32(0.45-3.90) |  | 0.311 | 1.77(0.59-5.34) |  | 0.459 | 1.48(0.52-4.18) |  | 0.263 | 1.46(0.75-2.81) |

**Note:** Wild, heterozygous, mutation, dominance model and recessive model of mTOR rs1064261 polymorphisms are TT, TC, CC, TC+CC vs. TT and CC vs. TC+TT, respectively. Wild, heterozygous, mutation, dominance model and recessive model of AKT rs1130233 polymorphisms are GG, GA, AA, GA+AA vs. GG, AA vs. GA+GG, respectively.

**Abbreviations:** HR, hazard rate; CI, confidence interval; NA, not available; T1+T2, mucosa, submucosa, muscularis propria; T3+T4, subserosa, serosa.
